# Metabolic and behavioral features of acute hyperpurinergia and the maternal immune activation mouse model of autism spectrum disorder

**DOI:** 10.1371/journal.pone.0248771

**Published:** 2021-03-18

**Authors:** Zarazuela Zolkipli-Cunningham, Jane C. Naviaux, Tomohiro Nakayama, Charlotte M. Hirsch, Jonathan M. Monk, Kefeng Li, Lin Wang, Thuy P. Le, Simone Meinardi, Donald R. Blake, Robert K. Naviaux

**Affiliations:** 1 The Mitochondrial and Metabolic Disease Center, University of California, San Diego School of Medicine, San Diego, CA, United States of America; 2 Department of Neurosciences, University of California, San Diego School of Medicine, San Diego, CA, United States of America; 3 Department of Chemistry, University of California, Irvine (UCI), Irvine, CA, United States of America; 4 Department of Medicine, University of California, San Diego School of Medicine, San Diego, CA, United States of America; 5 Department of Pediatrics, University of California, San Diego School of Medicine, San Diego, CA, United States of America; 6 Department of Pathology, University of California, San Diego School of Medicine, San Diego, CA, United States of America; California State Polytechnic University Pomona, UNITED STATES

## Abstract

Since 2012, studies in mice, rats, and humans have suggested that abnormalities in purinergic signaling may be a final common pathway for many genetic and environmental causes of autism spectrum disorder (ASD). The current study in mice was conducted to characterize the bioenergetic, metabolomic, breathomic, and behavioral features of acute hyperpurinergia triggered by systemic injection of the purinergic agonist and danger signal, extracellular ATP (eATP). Responses were studied in C57BL/6J mice in the maternal immune activation (MIA) model and controls. Basal metabolic rates and locomotor activity were measured in CLAMS cages. Plasma metabolomics measured 401 metabolites. Breathomics measured 98 volatile organic compounds. Intraperitoneal eATP dropped basal metabolic rate measured by whole body oxygen consumption by 74% ± 6% (mean ± SEM) and rectal temperature by 6.2˚ ± 0.3˚C in 30 minutes. Over 200 metabolites from 37 different biochemical pathways where changed. Breathomics showed an increase in exhaled carbon monoxide, dimethylsulfide, and isoprene. Metabolomics revealed an acute increase in lactate, citrate, purines, urea, dopamine, eicosanoids, microbiome metabolites, oxidized glutathione, thiamine, niacinamide, and pyridoxic acid, and decreased folate-methylation-1-carbon intermediates, amino acids, short and medium chain acyl-carnitines, phospholipids, ceramides, sphingomyelins, cholesterol, bile acids, and vitamin D similar to some children with ASD. MIA animals were hypersensitive to postnatal exposure to eATP or poly(IC), which produced a rebound increase in body temperature that lasted several weeks before returning to baseline. Acute hyperpurinergia produced metabolic and behavioral changes in mice. The behaviors and metabolic changes produced by ATP injection were associated with mitochondrial functional changes that were profound but reversible.

## Introduction

Over the past decade our group has tested a new unifying hypothesis for the origin and treatment of autism spectrum disorder (ASD) in both environmental and genetic animal models [1–3] and a small human clinical trial [4]. This unifying hypothesis proposes that the behavioral symptoms and neurobiology of ASD are the result of a metabolic syndrome that arises from abnormalities in purinergic signaling. Abnormalities in purinergic signaling can be produced by genetic or environmental changes, or by the interaction of both. Purinergic signaling was first described by Geoffrey Burnstock in 1970 [5], and refers to the action of purines like ATP, ADP, AMP, and adenosine, and some pyrimidines like UTP and UDP-glucose, when they bind to specific cellular receptors [6]. Of note, ATP or its metabolites have also been found to be co-neurotransmitters and neuromodulators at every synapse in the central and enteric nervous systems, and every immunologic synapse that has been studied to date [7–10].

In recent years, it has been found that all stressed cells release ATP in proportion to the degree of environmental threat through stress- and redox-gated pannexin/P2X7 and other channels in the plasma membrane, and by vesicular export [11, 12]. The stress can come in the form of either positive or negative threats. Positive threats refer to the presence of or exposure to noxious agents like infection, pollution, genotoxicity, physical or psychological trauma. Negative threats refer to the absence of or deficiencies in needed resources like oxygen, water, calories, vitamins, nutrients, social interaction [13], or even gravity [14]. Each of these stresses produces functional changes in mitochondria and leads to an increase in cellular ATP release. Once released, the extracellular ATP then serves as a pro-inflammatory signal and damage associated molecular pattern (DAMP) [15, 16] that is an effector of the cell danger response (CDR) [17]. The leakage of ATP to the extracellular space also has the effect of decreasing intracellular ATP pools and energy reserves, prompting further adaptive changes in mitochondria, metabolism, and gene expression.

Mitochondria coordinate the response to cellular stress by producing 90% of the ATP in the cell, and by regulating cellular redox, energy metabolism, and epigenetics [18, 19]. Mitochondria also play a pivotal role in both the response to [20], and regeneration after injury [21]. Independent studies around the world have found abnormalities in purinergic signaling in autism spectrum disorder (ASD). Gene ontology analysis of transcriptomic data from post-mortem brains of children with ASD found that purinergic signaling abnormalities correlated with ASD behaviors [22]. A metabolomic study of children with ASD in Italy concluded that their findings were consistent with a ‘purine-driven cell danger response’ [23]. Other metabolic markers of the cell danger response, like changes in tryptophan, methionine, folate, and glutathione metabolism, and changes in the microbiome [24] have been described in several cohorts of children with ASD and have been shown to trace to a new state of mitochondrial function [25, 26].

The first genetic evidence that abnormalities in purinergic signaling might be involved in the pathogenesis of ASD came in 1969 [27]. This was the report of a child with ASD and high uric acid from increased purine metabolism. In this child a mutation in phosphoribosyl pyrophosphate synthase (PRPPS) eliminated feedback inhibition and led to superactivity of this rate-limiting enzyme in *de novo* purine synthesis [28]. Other studies soon followed that reported subsets of children with ASD and mitochondrial abnormalities associated with increased lactic acid and others with purine synthesis and uric acid abnormalities [29–32]. Several other examples of genetic disorders of purine and pyrimidine metabolism that cause ASD have been reported [33]. The first mitochondrial DNA mutation to cause ASD was reported in 2000 [34]. Based on these genetic leads, we tested the antipurinergic drug suramin to treat the Fragile X genetic model of autism in mice. That study found the top metabolic pathway that changed in association with the correction of ASD-like behaviors was purines [1]. Independent molecular support for the CDR hypothesis and the role of purinergic signaling in ASD has come from recent studies in Fragile X syndrome that have shown that ATP leak from mitochondria and cells is directly related to neurobiological abnormalities [35]. In the current mouse study, we used a combination of bioenergetic, breathomic, metabolomic, and behavioral analysis to address two aims: 1) to test the effect of ATP injection in typically developing control animals as an experimental model of hyperpurinergia, and 2) to test the effects of postnatal ATP or poly(IC) injection in the maternal immune activation (MIA) model [36–38].

## Materials and methods

### Animals and husbandry

All studies were conducted in facilities accredited by the Association for Assessment and Accreditation of Laboratory Animal Care International (AAALAC) under UCSD IACUC-approved animal subjects protocol number S06135. C57BL/6J mice were obtained from Jackson Laboratories (Bar Harbor, ME) and maintained on *ad libitum* Harlan Teklad 8604 mouse chow and water. Animals were housed at an ambient temperature of 22–24˚C and humidity of 40–50%, in a controlled access vivarium with a 12h light-dark cycle; lights on at 7 am and off at 7 pm. The thermoneutral zone for adult mice about 25–30 grams in weight is 29–31˚C [39]. Below this temperature, additional calories must be burned to maintain body temperature. The human biological age equivalent for the C57BL/6J strain of laboratory mouse (*Mus musculus*) was estimated from the following equation: 12 years for the 1^st^ month, 6 years for the 2^nd^ month, 3 years for months 3–6, and 2.5 years for each month thereafter [2, 40]. Therefore, an 8-month old mouse would be about 35 years old (= 12 + 6 + 3*4 + 2.5*2 = 35 years) on a human timeline. Experimental treatment groups included animals from at least two different litters to mitigate against behavioral and metabolic litter effects. No mice were sacrificed as part of this study.

### Reagents

ATP, ADP, GTP, adenosine (Ado), cAMP, cGMP, UTP, CTP, TTP, and poly(IC) were purchased from Sigma. Sterile saline was made from endotoxin-free, nuclease free H_2_O and used as the solvent for all solutions. The sodium salt of each nucleotide was used when available. When only the free acid form was available an aqueous solution of 100–200 mM was first prepared. This was then neutralized with 5M NaOH to pH 7.0 and diluted to the desired stock concentration and frozen until use.

### Nucleotide administration

All nucleotide and drug challenge experiments were conducted between 9:00 am and 1 pm. Mice were given intraperitoneal (i.p.) doses of nucleotides or saline at an equal volume of 20 μl/g. i.p. doses up to 0.5 μmoles/g were given as a 25 mM solution. Intravenous (i.v.) doses were administered by lateral tail vein injection in maximum volumes of 5 μl/g. i.v. doses up to 0.5 μmoles/g were given as a 100 mM solution to minimize intravascular volume effects. Pilot dose-response experiments revealed that higher doses (>0.5 μmoles/g (>0.25 g/kg), as 50–100 mM solutions) of ATP i.p. resulted in non-linear phenotypic dose responses as they approached saturation. To initiate the maternal immune activation (MIA) model, pregnant dams received an i.p. injection of poly(IC) (Sigma-Aldrich Cat# P9582;) (0.17 A260 U/g; 2 mg/kg i.p.) on gestational day E12.5 and E17.5. Control dams received normal saline (0.15 M NaCl; 5 μl/g i.p.) on E12.5 and E17.5. Pregnant dams were provided with nesting material and left undisturbed until offspring were weaned at 3–4 weeks of age. The postnatal challenge dose of poly(IC) (0.17 A260 U/g; 2 mg/kg i.p.) was tested in 8-9-month-old animals. Daily temperature was recorded for 14 days, then weekly for 4 weeks after poly(IC) administration.

### Metabolomics

Blood was collected by submandibular vein lancet [41] into lithium-heparin BD microtainers (Cat# 365971, Becton-Dickinson) and inverted 10 times. Plasma was separated by centrifugation at 1500 g for 5 min in an Eppendorf microfuge and frozen at -80˚C until use. Blood draws were performed between the hours of 9 am and 1 pm. Targeted, broad-spectrum, metabolomic analysis of 613 metabolites from 63 biochemical pathways was performed by LC-MS/MS as described [42]. A total of 401 of the 613 targeted metabolites were measurable in the plasma of both males and females. Targeted metabolites were identified by retention times and multiple reaction monitoring (MRM) of precursor and product ion transition pairs and confirmed using one of over 700 purified compounds and stable isotope labeled standards in our in-house chemical inventory. There were no missing values, and no imputation was used. L-cysteine was not measured independently because of oxidation to its disulfide cystine that occurred in whole plasma with storage.

### Breathomics

Breathomic analysis of natural gases and volatile organic compounds in mice was performed as described [43] with minor modifications as described below. Six 10-month old C57BL/6J male mice were used in this study. Three mice received i.p. injections with physiologic saline, and 3 were injected with 0.5 μmol/g ATP (0.6 ml i.p. of a 25 mM solution in 30-gram animals). Samples of exhaled mouse breath were collected from a 10 L glass bulb containing a mouse fitted with three sampling ports. One of the sampling ports was connected to a 1.9 L electropolished stainless steel canister via a Swagelok Nupro bellows valve using stainless steel flex tubing. Prior to sampling, the canisters were baked at 150°C for 24 hours, flushed with ultra-high purity helium, and vacuumed to 10^−2^ mm Hg. Upon injection, each mouse was immediately placed in the sampling vessel and breath samples were collected for 20 seconds at 1, 5 and 10-minute intervals into three separate 1.9 L canisters for each mouse. For purposes of this analysis, the results for all 3 time points were pooled and averaged. Room air samples were collected before and after the study for background air analysis. The samples were analyzed for CO/CO_2_ and CH_4_ in addition to a list of about 100 volatile organic compounds (VOCs) using the 6-column-detector gas chromatography system in the Rowland/Blake laboratory at UC Irvine. The CO/CO_2_ measurements were carried out using a Carbosphere 80/100 packed column with a flame ionization detector (FID) for CO and a thermal conductivity detector (TCD) for CO_2_. CH_4_ determination was performed on a separate GC system consisting of a packed column terminating at an FID. Analysis on the multi-column system began with cryogenic pre-concentration of sample at 400 mm Hg, followed by injection into 6 separate columns, contained in pairs in a combination of 3 ovens (Hewlett-Packard 6890 series GC system). Temperature ramp programs were set at -60°C to 220°C for 2 ovens and -20°C to 200°C for the third. This GC system utilized several detectors including FID, electron capture (ECD), and mass selective detectors (MSD). The combination of columns and detectors includes DB-1/FID (Agilent), PLOT+DB-1/FID (Agilent), Restek1701/ECD (Restek), DB-5+Restek1701/ECD (Agilent, Restek) and DB-5ms/MSD (Agilent), many of which quantify the same gases. The redundant gases for each column were plotted against one another, to ensure a linear trend. This elucidated chemical differences of any co-eluting peaks by comparison of different column results. Exhaled hydrogen sulfide (H_2_S) or methanethiol (CH_4_S, also called monomethylsulfide) was not quantified in this analysis.

### Chemokines and cytokines

The plasma cytokine response to i.p. ATP (0.5 μmol/g) or saline was examined at baseline, 30-minutes and 4-hours after injection in 6-month old female C57BL/6J mice. Heparinized plasma was diluted 2-fold in saline and analyzed at the UCSD CTF-C Core Lab using the 7-plex MSD Multispot Assay system (Meso Scale Diagnostics, LLC, Gaithersburg, MD, USA) according to manufacturer instructions. The 7 cytokines examined and the lower limit of detection (LLOD in pg/ml) were: IL10 (11), CXCL1 (3.3), IL12p70 (35), IL1β (0.75), TNFα (0.85), IL6 (4.5), and IFNγ (0.38).

### Corticosterone assays

Four-month old C57BL/6J females (N = 7 per time point) were treated with 0.5 μg/g ATP i.p. or saline at time 0. Blood samples were collected before, 30-minutes after, and 4-hours after treatment. Plasma concentrations of corticosterone were measured by ^125^I double antibody radioimmunoassay using 10 μl of plasma diluted 1:200 with assay buffer (MP Biomedicals LLC, Orangeburg, NY). The intraassay coefficient of variation (CV) was 4% with an interassay CV of 7%.

### Temperature measurements

A BAT-12 Microprobe digital thermometer and RET-3 mouse rectal probe (Physitemp Instruments, Clifton, New Jersey) were used to obtain a temperature precision of +/-0.1°C, taking care to minimize stress-induced hyperthermia, as previously described [3]. Body temperature was used as a marker for associated changes in mitochondrial bioenergetics and metabolism [44, 45].

### Basal metabolic rate

Food intake, oxygen consumption and locomotor activity were measured using the Comprehensive Lab Animal Monitoring System (CLAMS, Columbus Instruments, Columbus, OH) in 13-minute cycles over a 3-hour period after nucleotide injection. Each mouse was housed in individual CLAMS cages with ad libitum access to Teklad 8604 standard mouse chow and water. The respiratory exchange ratio was computed as the rate of CO_2_ production divided by the rate of oxygen consumption (RER = VCO_2_/ VO_2_).

### Behavior

ATP injection produced rapid behavioral changes that started within minutes and returned to baseline in 30–90 minutes, depending on the dose and route (i.p. or i.v.) of administered nucleotide. These rapid kinetics could not be accurately captured by conventional behavioral measures like social approach and T-maze that we had previously used in our MIA and Fragile X mouse studies because they take hours to complete [1–3]. To meet the kinetic challenge, we developed a semi-quantitative purinergic behavioral response scale (PBRS) to measure the observed stereotyped behavioral and physiologic responses to purines and pyrimidines. The PBRS was a rapid 3-point severity scale (0-absent, 1-present, and 2-present and severe) that assessed 6 characteristics: 1) cage center avoidance observed when injected animals were placed in the center of the cage and would move spontaneously to an edge or corner, 2) decreased locomotor activity or complete stillness, 3) imbalance/ataxia in a cage rim walk test, 4) generalized piloerection, 5) rapid shallow breathing/panting, 6) shivering/rigors. The PBRS range was 0 (normal) to 12 and was performed in the home cage. PBRS assessments were performed at 0, 15, 30 and 60 minutes after ATP injection.

### Statistical analysis

Results are reported as mean ± SD unless otherwise noted. Peak area under the curve (AUC) data from metabolomics were log_2_ transformed, scaled by control standard deviations, and the resulting z-scores analyzed by univariate non-parametric analysis by Mann-Whitney U test, and parametric analysis of ≥ 3 groups by ANOVA. Cytokine data were log_2_ transformed using the generalized log transformation formula glog_2_ = log_2_(y+1) to accommodate fractional concentrations between 0 and 1 pg/ml. Simple linear regression was used to compare dose-dependent rates of temperature decline. Linear mixed models analysis was used for longitudinal time and temperature data. Metabolomic data were analyzed by multivariate partial least squares discriminant analysis (PLSDA). Post hoc correction for multiple hypothesis testing after ANOVA was done by Fisher’s least significant difference method in MetaboAnalyst [46, 47]. The false discovery rate (FDR) method of Benjamini and Hochberg [48] was used for all other analyses. Bayesian false discovery rates were estimated using the Storey q value [49]. Metabolites with variable importance in projection (VIP) scores were determined by PLSDA. Significant metabolites were grouped into pathways and their VIP scores summed to determine the rank-ordered significance of each biochemical pathway. Random forest analysis [50] was used to rank metabolites for their ability to distinguish the different treatment groups using mean decrease in accuracy (MDA) scores. The k-nearest neighbor (k-NN) algorithm is a machine learning tool and non-parametric classification method that clusters variables that behave in similar ways. Its utility in metabolomics is that k-NN methods do not force metabolites into the same group just because they are in the same biochemical pathway. k-NN clustering was used to identify superclusters of metabolites that changed in coordinated ways after ATP injection but can come from many different biochemical pathways [51]. k-NN clusters were ranked by the sum of the VIP scores ≥1.0 and by positive MDA scores. Dendrograms were Euclidean using Ward clustering. Pearson and Spearman correlation and logistic multiple regression was used to identify metabolites most associated with acute ATP injection. Results were organized into biochemical pathways and visualized in Cytoscape version 3.4.0. Statistical methods were implemented in Stata (Stata/SE12.1, StataCorp, College Station, TX), Prism (Prism 8, GraphPad Software, La Jolla, CA), Python, or R.

## Results

### Study overview

**Fig 1:**
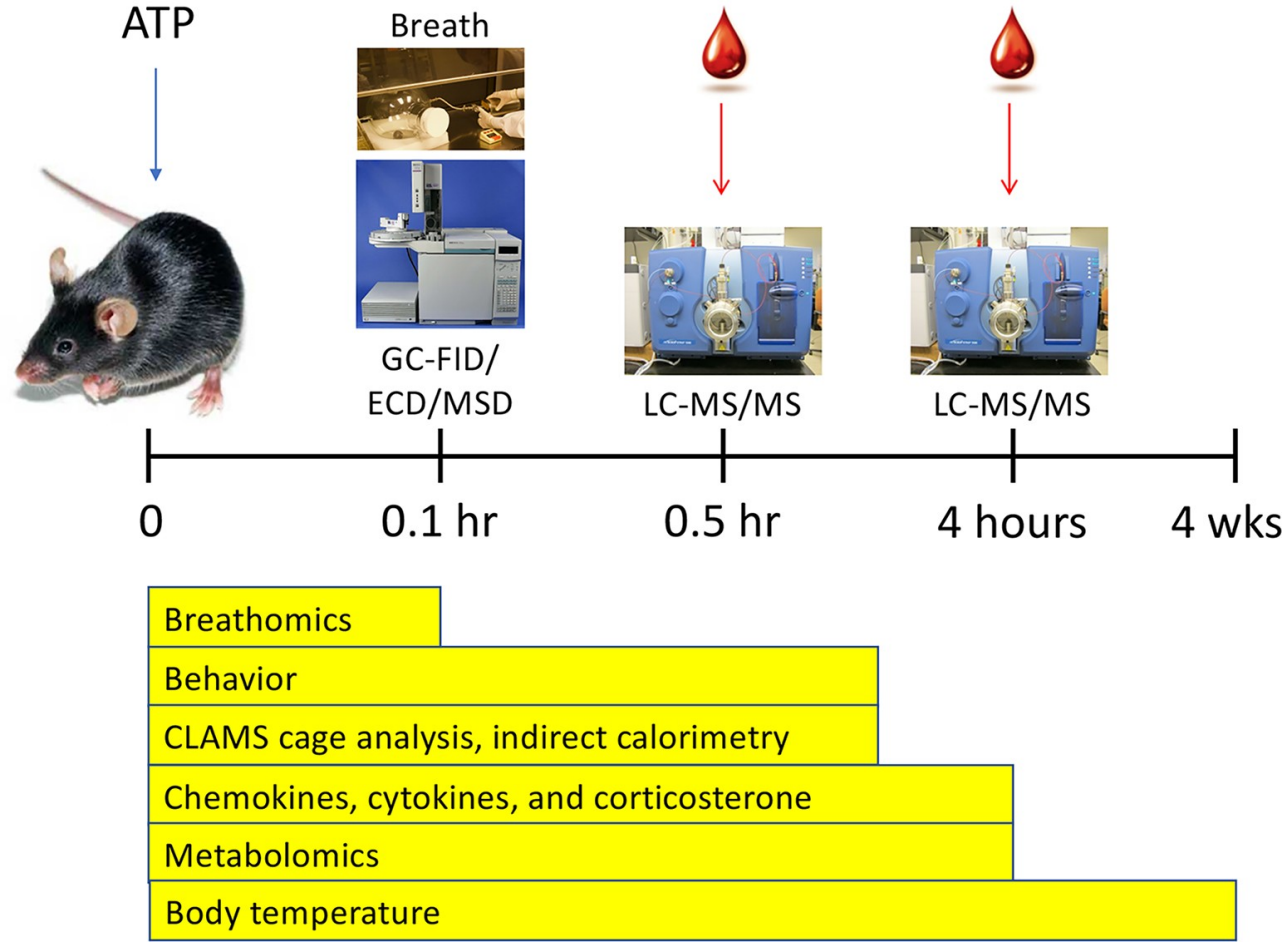
Study overview. Abbreviations: GC—gas chromatography, FID—flame ionization detection, ECD—electron capture detection, MSD—mass selective detection, LC—high performance liquid chromatography, MS/MS—triple quadrupole mass spectrometry.

The overall design of this study is illustrated in Fig 1. Six different experimental techniques were used to characterize the acute and subacute responses to ATP injection.

### Metabolomics

**Fig 2:**
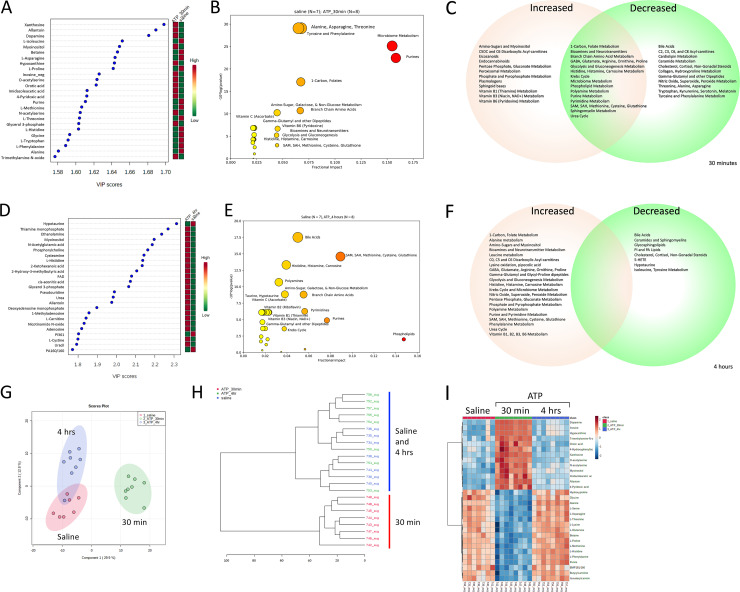
Metabolomic analysis of acute hyperpurinergia. A. Ranking of metabolites changed 30 minutes after ATP injection by partial least squares discriminant analysis (PLSDA). B. Bubble impact plot of pathways most changed 30 minutes after ATP injection, C. Venn diagram of pathways increased, decreased, or contained increased and decreased metabolites after 30 minutes, D. Ranking of metabolites changed 4 hours after ATP injection by partial least squares discriminant analysis (PLSDA), E. Bubble impact plot of pathways most changed 4 hours after ATP injection, F. Venn diagram of pathways and metabolites increased or decreased after 4 hours, G. Two-dimensional separation of the metabolomes by multivariate PLSDA components after saline and 0.5 and 4 hours post ATP injection, H. Dendrogram showing sharp separation of the metabolome at 30 minutes and the heterogeneous and incomplete return to baseline by 4 hours after ATP injection, I. Heatmap of the top 30 most-changed metabolites 30 minutes and 4 hours after ATP injection. ATP dose = 0.5 μmol/g body weight, n = 7–8 C57BL/6J males per group, age = 12–13 weeks. Abbreviations: VIP—variable importance in projection.

A total of 202 (50%) of 401 metabolites measured were significantly changed by ATP injection in 30 minutes (S1–S5 Tables, S1-S12 Figs in S1 File). The rank order of metabolites that were most changed is illustrated in Fig 2A. These metabolites belonged to 37 different biochemical pathways (Fig 2B and 2C, S3 Table) and showed FDRs <0.05, VIP ≥ 0.9, and t-test p values < 0.05 (S2 Table). There was a generalized decrease in plasma amino acids. All amino acids—both essential and non-essential–were affected (S3 Fig in S1 File). The average decrease across the 19 amino acids measured was -5.0 ± 2.6 (Z-score ± SD; S2 Table, S3 Fig in S1 File). This was equivalent to a mean decrease in amino acid AUCs of 41 ± 13%. Fifteen ceramides and a class of phospholipids enriched in lysosomal and exosomal membranes, the bis(monoacylglycero)phosphates (BMPs), were also decreased (S3 Table, S6 Fig in S1 File). Phosphatidylinositol (PI) lipids were decreased. The polar head groups of the major phospholipids were increased. This included choline, phosphorylcholine, ethanolamine and myoinositol. Myoinositol, which is a phospholipid head group derived by PI lipid activation to inositol phosphates for calcium signaling and subsequent processing by phosphatases, was sharply increased by ATP injection. Other head groups like phosphorylcholine and ethanolamine were also increased. Phosphatidylethanolamine (PE) lipids were decreased, while their precursors phosphatidylserine (PS) lipids were increased. Several arachidonate- and linoleate-derived signaling lipids, including 5-HETE and 13S-HODE, and the endocannabinoid anandamide, were also increased (S4 Fig in S1 File, S2 Table). Dopamine was strongly increased 30 minutes after ATP injection, consistent with its role as a multifunctional stress response effector [52] (Fig 2A, S2 Table). Other striking effects of acute ATP injection included an increase in lactate, glycerol-3-phosphate, associated with the expected increase in glycolysis that accompanies the observed decrease in mitochondrial oxidative phosphorylation. Pyrimidine precursors like orotic acid and products like beta-alanine, were also increased. As expected, purine metabolites were strongly increased (Fig 2A–2C, S2 and S9 Figs in S1 File). These included xanthosine, allantoin, inosine, hypoxanthine, xanthine, and uric acid. Allantoin was disproportionately increased compared to uric acid in the mouse because mice have an intact uricase gene, while primates do not [53]. Adenosine triphosphate (ATP) was rapidly metabolized and was not detectable in any of the plasma samples (S1 Table). An increase in the modified purine, 7-methylguanine derived by 5’ uncapping of mRNA, was consistent with a global decrease in cap-dependent cell translation and protein synthesis at 30 minutes (S2 Table, S2 Fig in S1 File).

The top 2 of 10 k-NN clusters at 30 minutes after ATP injection were responsible for 79% of the metabolic impact and contained 134 metabolites with VIP scores ≥ 1.0, from 36 different metabolic pathways. Seventy-one (71) metabolites were increased with a mean Z-score of +3.2 ± 1.8 (mean ± SD; k-NN cluster #2, S5 Table, S4 Fig in S1 File) and belonged to a superpathway that included purines but was comprised of 26 different biochemical pathways. Sixty-three (63) metabolites were decreased with a mean Z-score of -3.4 ± 2.0, including 19 of 19 measured amino acids, (k-NN cluster #1, S6 Table, S5 Fig in S1 File) and belonged to a second superpathway comprised of an overlapping set of 26 different biochemical pathways (S2–S5 Tables).

A total of 54 (13%) of 401 metabolites measured were changed at 4 hours after ATP injection (Fig 2D–2F, S1 and S7–S12 Tables, S1-S12 Figs in S1 File). After 4 hours of recovery, one of 8 animals had recovered sufficiently to be indistinguishable from controls by multivariate analysis, while most others had not recovered completely. This is seen graphically by the overlap of sample points and the 95% confidence limits shown in the 2D PLSDA plots (Fig 2G; blue and pink circles), and in the dendrograms that show a clean statistical separation of ATP- and saline-treated animals at 30 minutes, but an intermixed response after 4 hours (Fig 2H). During subacute recovery measured 4 hours after eATP injection, phospholipids and sphingolipids, were decreased. Several bile acids like glycocholic and taurocholic acid remained decreased. Increased turnover of phospholipids was evidenced by an increase in the phospholipid head groups phosphorylcholine, ethanolamine, and myoinositol. Several markers of cellular oxidation were increased 4 hours after ATP injection. These included increased oxidized glutathione (GS-SG) and cystine (CysS-SCys), and increased markers of carnosine metabolism such as 1-methylhistidine and histidine. Markers of increased mitochondrial tRNA turnover were also observed at this time. These included the post-transcriptionally modified purines and pyrimidines 1-methyladenosine and pseudouridine (S2 Fig in S1 File). Changes in nitrogen metabolism at 4 hours were marked by increases in N-acetylglutamate (NAG), agmatine, and urea (S4 Fig in S1 File).

The top 3 of 10 *k*-NN clusters at 4 hours after ATP injection were responsible for 88% of the metabolic impact and contained 45 metabolites with VIP scores ≥ 1.5 that belonged to 27 different metabolic pathways (S9–S11 Tables). Thirty-two (32) metabolites in the top k-NN cluster were increased with a mean Z-score of +2.2 ± 0.7 (mean ± SD; k-NN cluster #3, S10 Table) and belonged to a superpathway comprised of 19 different biochemical pathways. The second two superpathways contained 13 metabolites that were decreased with a Z-score range of -2.0 to -2.7 and were made up of 7 biochemical pathways (k-NN clusters #7 and #8, S9 and S11 Tables).

A heat map of the 30 most increased or decreased metabolites is shown in Fig 2I. The proportional effects of purinergic signaling on all the biochemical pathways measured at 30 minutes and 4 hours after injection are illustrated in the Cytoscape map in S1 Fig in S1 File. Quantitative changes in purines, amino acid, methylation, sulfur, polyamine, and nitrogen metabolism are illustrated in S9 Fig in S1 File. Principal components analysis showed that metabolomics explained 81.2% and 74.9% of the phenotypic variance in animals at 30 minutes and 4 hours after ATP injection, respectively (S10 Fig in S1 File).

Eleven products of microbiome metabolism increased 30 minutes after eATP injection (S7 Fig in S1 File). These included an increase in the cysteine precursor O-acetylserine, the leucine precursor isopropylmalic acid, the carnitine metabolite trimethylamine-oxide (TMAO), the histamine metabolite imidazoleacetic acid, two phenylketones from microbial tyrosine metabolism, 4-hydroxyphenyllactic acid and 4-hydroxyphenylpyruvic acid, and the aryl hydrocarbon receptor-binding immunomodulatory molecule and tryptophan metabolite indoxyl-3-sulfate [54]. The mean Z-score for increased microbiome metabolites in the plasma was +3.5 ± 1.5 (S2 Table). Only two microbiome metabolites, butyrylcarnitine (Z = -5.7) and vitamin K2 (menaquinone; Z = -1.2) were decreased 30 minutes after ATP injection. Unsubstituted purine, thought to be a marker of purine-rich food intake [55], was decreased (Z = -5.2) 30 min after ATP. The significance of this is not yet understood. No microbiome metabolites were abnormal 4 hours after ATP injection, although glycocholic, taurocholic, and taurodeoxycholic bile acids remained low (Z = -1.5 ± 0.2; S6 Table).

A broad range of vitamins were acutely changed in the plasma 30 minutes after ATP injection (S8 Fig in S1 File). Thiamine (B1), niacin (B3), pyridoxic acid (B6), and choline were increased by a mean Z-score of +3.4 ± 1.6 (S2 Table). In contrast, the plasma intermediates and effectors of 1-carbon metabolism were decreased. These included serine, glycine, and trimethyl-glycine (betaine), with a mean Z-score of -4.4 ± 1.8 (S2 Table). Vitamin D3 (cholecalciferol) was also decreased (Z-score = -1.5), although active 1,25-dihydroxy Vitamin D3 was unchanged (S2 Table). Thiamine (B1), niacin (B3), and pyridoxic acid (B6) remained increased in the plasma 4 hours after eATP injection, with a mean Z-score of +1.8 ± 1.1 (S6 Table, S8 Fig in S1 File). Other vitamins, cofactors that were increased at 4 hours included 5-methyl tetrahydrofolic acid (mTHF), dimethylglycine, flavin adenine dinucleotide (FAD; B2), and L-carnitine with a mean Z-score of +1.7 ± 0.9. No vitamins were decreased 4 hours after ATP injection (S6 and S9 Tables).

### Breathomics

**Fig 3:**
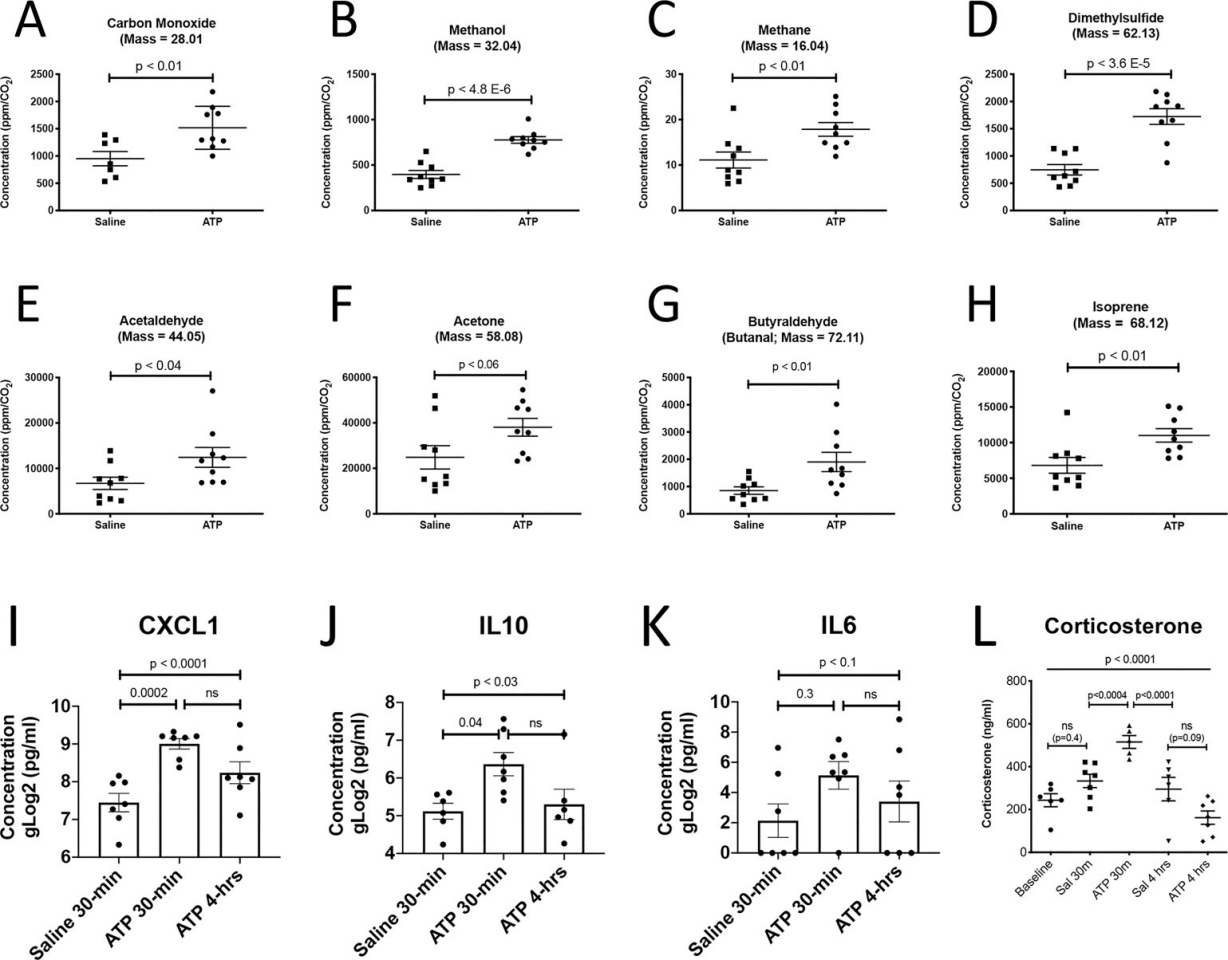
Breathomics, chemokines, cytokines, and corticosterone response to acute hyperpurinergia. Breathomics captured and analyzed exhaled breath at 1–10 minutes after ATP injection (A-H; n = 3 C57BL/6J males per group, 3 samples per animal), A. Carbon monoxide, B. Methanol, C. Methane, D. Dimethylsulfide, E. Acetaldehyde, F. Acetone, G. Butyraldehyde, H. Isoprene. Plasma chemokine and cytokine analysis 30 min and 4 hours after ATP injection (I-K; n = 6–7 C57BL/6J males per group), I. CXCL1/KC/GRO J. IL10, K. IL6. Plasma corticosterone levels 30 minutes and 4 hours after ATP injection (ATP dose = 0.5 μmol/g body weight, n = 7 C57BL/6J females per group), L. Corticosterone. Abbreviations: CXCL1—chemokine (C-X-C motif) ligand 1, KC—keratinocyte-derived chemokine, GRO—growth related oncogene alpha, IL10—interleukin 10, IL6—interleukin 6. P-values: * = 0.05, ** = 0.01, *** = 0.001, **** = 0.0001.

Accurate measurements of exhaled gases requires normalization for minute volumes using the rate of CO_2_ production [43]. We found that ATP injection stimulated the release of volatile organic molecules ranging from 1 to 5 carbons in length in the first 10 minutes (Fig 3A–3H). These included the three different 1-carbon species: carbon monoxide (CO), methanol, and methane. One 2-carbon, sulfur-containing volatile was increased by ATP injection: dimethylsulfide. The remaining volatiles that were produced by acute hyperpurinergia included acetaldehyde (C2), acetone (C3), butyraldehyde (C4), and isoprene (C5; Fig 3A–3H, and S12 Fig in S1 File).

### Chemokines and cytokines

Cytokines were measured at baseline, 30-minutes and 4-hours after ATP or saline injection to permit comparison of metabolomic and cytokine data at these time points. The chemokine CXCL1, also known as KC and GROα, was increased 2.8 times compared to saline injections (526 ± 118 pg/ml vs 188 ± 75; p < 0.0002). CXCL1 binds the G-protein coupled receptor CXCR2 and facilitates the arrest of rolling neutrophils and monocytes at sites of inflammation [56]. The anti-inflammatory interleukin, IL10 was also increased (95 ± 56 vs 36 ± 12; p < 0.04) (Figs 3I–3K). ATP was known to stimulate IL10 secretion from microglial cells in culture [57], but had not been studied in animals. By 4 hours, each of these had returned to baseline levels. IL6 trended toward being increased at 30-minutes but animal-to-animal variability in the saline controls limited a stronger statistical conclusion without a larger sample size (Fig 3K). IL1β, TNFα, IFNγ, and IL12p40 were measured and were unchanged at 30 minutes and 4 hours after i.p. ATP injection.

### eATP effects on corticosterone release

Previous studies have shown that adrenal corticoid synthesis and release are directly stimulated by purinergic signaling at the adrenal cortex, independent of ACTH [58]. We found that plasma corticosterone peaked 30 minutes after injection of ATP, then trended below baseline levels by 4 hours (Fig 3L). This pattern of response was consistent with acute stimulation of corticosterone release, followed by feedback inhibition of hypothalamic corticotropin releasing hormone (CRH) and ACTH. CRH and ACTH levels were not measured in this study.

### eATP effects on body temperature

**Fig 4:**
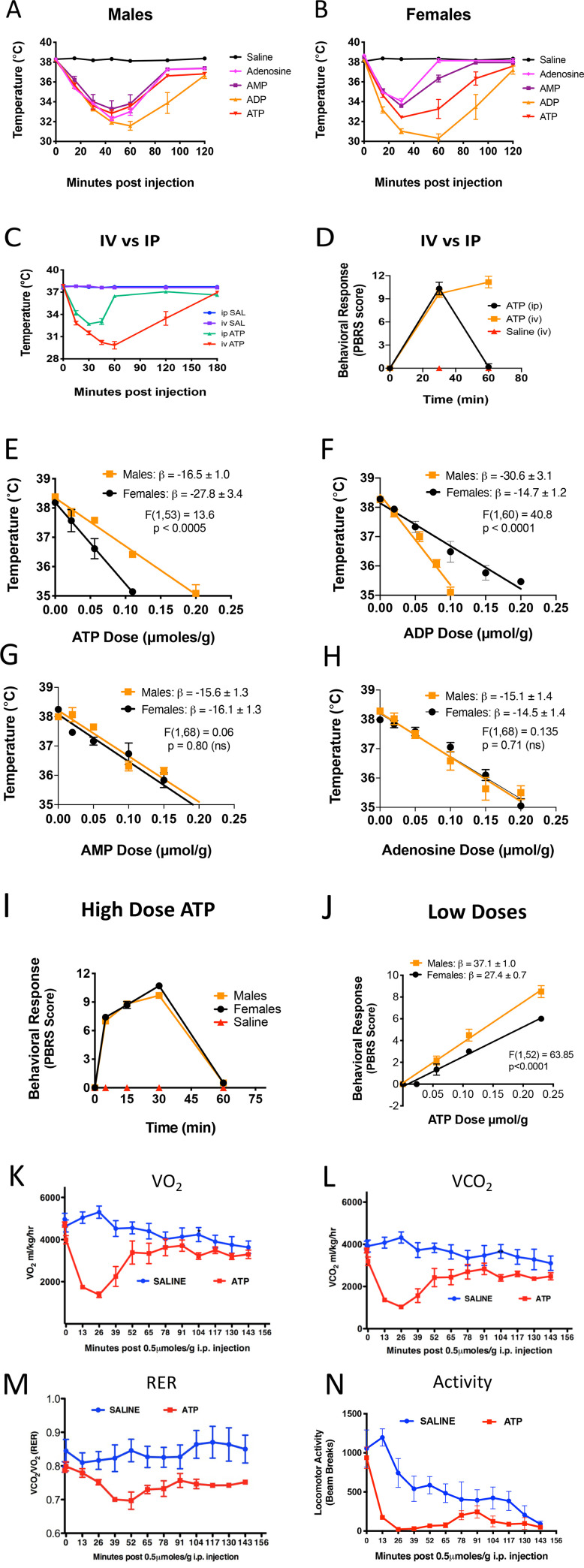
Body temperature, bioenergetic, and behavioral responses to acute hyperpurinergia. A. Male C57BL/6J mice (nucleotide dose = 0.5 μmol/g body weight, n = 6 per group, 5–6 months old), B. Female C57BL/6J mice (n = 6 per group, 5–6 months old). Intravenous (i.v.) vs intraperitoneal (i.p.) dosing (C and D, ATP dose = 0.5 μmol/g, n = 6 females per group, 5 months old), C. Body temperature response, D. Behavioral response. Sex-specific differences (E-H, temperatures measured at 15 minutes post-injection with 0–0.2 μmol/g ATP, n = 6–8 mice/group) E. Females were more sensitive to the hypothermic effects of ATP, F. Males were more sensitive to the hypothermic effects of ADP, G. Males and females were equally sensitive to the hypothermic effects of AMP, H. Males and females were equally sensitive to the hypothermic effects of adenosine. Behavioral responses (I and J), I. The behavioral response to high-dose ATP was the same in males and females (dose = 0.5 μmol/g, n = 10 per group), J. Dose-response curves at non-saturating ATP doses revealed that males were more sensitive to the behavioral effects of hyperpurinergia (PBRS scores measured at 15 minutes post-ATP, n = 6 per group, 5–6 months old). CLAMS cage analysis of bioenergetics (K-N, ATP dose = 0.5 μmol/g, n = 6 per group, 28-week old C57BL/6J females), K. The basal metabolic rate measured as the rate of oxygen utilization (VO_2_) was decreased by 74% after ATP injection, L. The rate of CO_2_ production was decreased by 76% after ATP injection, M. The respiratory exchange ratio (RER) dropped from 0.84 to 0.70 after ATP injection compared to saline, N. ATP injection decreased locomotor activity as measured by light beam breaks compared to saline. Abbreviations: PBRS—purinergic behavioral response scale, SAL—saline, CLAMS—comprehensive laboratory animal monitoring system. RER—respiratory exchange ratio = VCO_2_/VO_2_. Ambient temperature for all experiments was 22˚-24˚C.

We tested several nucleotides for their hypometabolic effects at the high dose of 0.5 μmol/g i.p. in both males and females (Fig 4A and 4B, S10 Fig in S1 File). All adenine-containing purines (adenosine, AMP, ADP, and ATP) produced a decrease in rectal temperature with a nadir that was reached 30–60 minutes after injection and recovery by 120 minutes. This effect lasted longer when the dose was administered intravenously instead of i.p. (Fig 4C). The behavioral changes caused by ATP also lasted longer when given i.v. (Fig 4D). ADP was most potent at these high doses of 0.5 μmol/g i.p. in both males and females (Fig 4A and 4B). We next evaluated the sex-specific potency of each purine under non-saturating doses of 0 to 0.20 μmol/g measured at 15 minutes to reflect the initial phase of the metabolic response. Under these conditions we found that females were about 70% more sensitive to the hypothermic effects of ATP, i.e., had a more rapid decrease in temperature (Fig 4E, Table 1), while males were more than twice (108%) as sensitive to ADP (Fig 4F, Table 1). AMP and adenosine were equally potent in both males and females (Fig 4G and 4H). We also examined the metabolic effects of several other purines and pyrimidines and cyclic nucleotides at equimolar doses of 0.5 μmol/g i.p. compared to saline and ATP (S11 Fig in S1 File). In males, only cAMP showed a hypometabolic effect similar to ATP. In females, both cAMP and GTP showed some activity, but both were less potent than ATP.

**Table 1:** Metabolic and behavioral features of acute hyperpurinergia.

Phenotype	Parameter (26–30 min post-infusion)	Saline Response (mean)	SD	ATP Response (mean)	SD	Change	Animals Per Group	p value
**Metabolic**^**1**^	Oxygen consumption (VO_2_ ml/kg/hr)	5302	710	1382	325	-74%	6	0.0001
	CO_2_ production (VCO_2_ ml/kg/hr)	4324	647	1034	226	-76%	6	0.0001
	RER (VCO_2_/VO_2_)^2^	0.84	0.082	0.70	0.062	-0.14	6	0.006
	Radiated heat (cal/hour)	650	84	180	40	-72%	6	0.0001
	Locomotor activity (xyz photobeam breaks)	742	442	27	16	-96%	6	0.006
**Core Temp**	Males (˚C)^3^	37.9	0.38	34.3	0.99	-3.6˚C	6	0.0005
	Females (˚C)^3^	38.4	0.13	32.2	0.26	-6.2˚C	6	0.0001
	Male dose response (β = ˚C/μmol/g ATP)^4^	0	0	-16.5	1.0	n/a	6	0.0005^4^
	Female dose response (β = ˚C/μmol/g ATP)^4^	0	0	-27.8	3.4	n/a	6	0.0005^4^
**Behavior**^**5**^	Males, maximal response (after 0.5 μmol/g)	0	0	9.7	0.48	n/a	10	0.0001
	Females, max response (after 0.5 μmol/g)	0	0	10.7	0.48	n/a	10	0.0001
	Male dose response (β = PBRS/μmol/g ATP)^6^	0	0	37.1	1.0	n/a	6	0.0001^6^
	Female dose response (β = PBRS/μmol/g ATP)^6^	0	0	27.4	0.7	n/a	6	0.0001^6^

^1^ATP Dose = 0.5 μmol/g i.p. C57BL/6J females, 28 weeks old in CLAMS cages.

^2^Maximum change in RER was not reached for 52 minutes.

^3^Rectal temperature. 20-week old C57BL/6J. Cage temperature = 22.4˚C, ATP dose = 0.5 μmol/g i.p. Males reached maximum hypothermia at 30 minutes, and females reached maximum at 45 minutes after injection.

^4^Linear regression analysis comparing the slopes (β) for males and females: F(1,53) = 13.6, p < 0.0005.

^5^Purinergic Behavioral Response Scale (PBRS).

^6^Linear regression analysis of the non-saturating dose-response to ATP scored at 15 minutes, comparing the slopes (β) for males and females: F(1,52) = 63.85, p < 0.0001.

### eATP effects on behavior

Intraperitoneal injection of ATP produced rapid behavioral changes that started within 1–2 minutes, peaked at 30 minutes, and resolved by 60 minutes. Behavioral changes included a rapid avoidance of the center of the cage, decreased locomotor and exploratory activity, and gait coordination abnormalities (Fig 4I). Normal movements gradually reappeared after 45–60 minutes, but the abnormal behavioral features were prolonged after an intravenous (i.v.) dose of ATP (Fig 4D). When non-saturating, low doses of ATP were given i.p. (0.025–0.20 μmoles/g; Fig 4J), and outcomes were measured in the linear initial phase 15 minutes after injection, significant sex differences were observed in the response to extracellular ATP (eATP injection). Males were 37% ± 3% more sensitive to the behavioral effects produced by eATP than females (male behavioral response slope β = 37.1 ± 1; female β = 27.4 ± 0.7; p<000.1; Fig 4J, Table 1).

### eATP effects on whole-body mitochondrial function

The effects of eATP injection on whole body metabolism, bioenergetics, and locomotor activity were quantified in Comprehensive Lab Animal Monitoring System (CLAMS cages, Fig 4K–4N). By 26 minutes after a dose of 0.5 μmol/g ATP, whole body oxygen consumption (VO_2_) dropped by 74% ± 6% (5,303 to 1,382 ml/kg/hr, p<0.0001; Fig 4K) and the rate of CO_2_ production (VCO_2_) dropped by 76% ± 18% (4323 to 1034 ml/kg/hr, p<0.0001; Fig 4L). The respiratory exchange ratio (RER = VO_2_/VCO_2_) shifted from 0.84 ± 0.08, reflecting a balanced usage of fat and carbohydrate to nearly complete dependence on fatty acids with an RER = 0.70 ± 0.062, p<0.006; Fig 4M, Table 1). Locomotor activity declined in both saline and ATP injected animals when placed in the wire-bottomed CLAMS cages for analysis, but the ATP-injected animals were nearly motionless between 26–52 minutes (Fig 4N, Table 1).

### The MIA model

**Fig 5:**
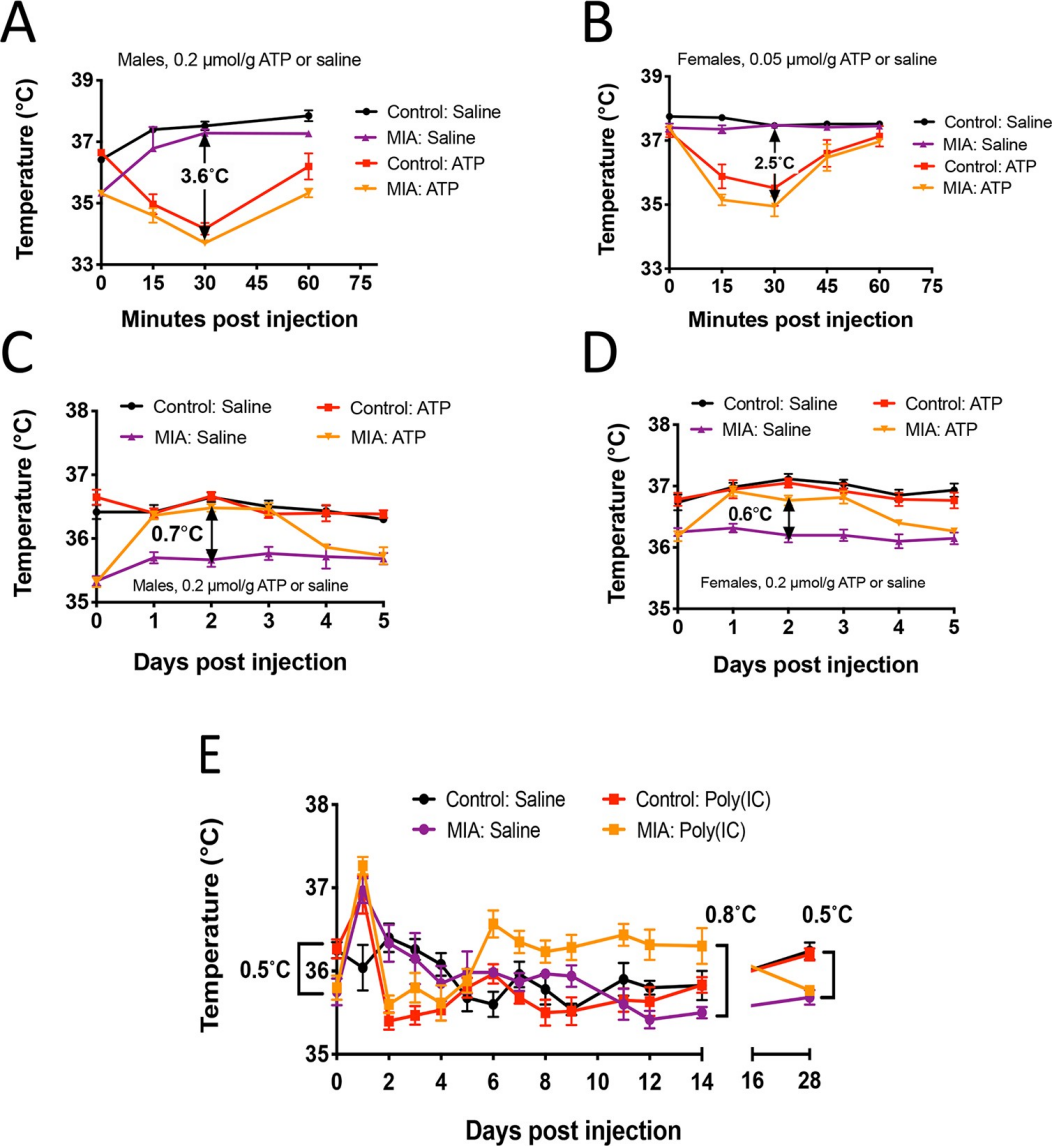
Thermoregulation and the latent memory response to ATP and poly(IC) in the MIA mouse model. The acute 1-hour response to postnatal challenge with ATP (A and B, n = 6 per group) A. Acute response in males, B. Acute response in females. The subacute 5-day response to postnatal challenge with ATP (C and D, n = 6 per group, 8–9 months old) C. Five-day response to ATP in males, D. Five-day response to ATP in females, E. The triphasic temperature response to postnatal challenge with poly(IC) (dose = 2 mg/kg, n = 6 males per group, 8–9 months old). Abbreviations: MIA—maternal immune activation mouse model, Poly(IC)—poly inosinic:cytosinic acid double strand RNA. Ambient temperature for all experiments was 22˚-24˚C.

In the MIA model, pregnant female mice are exposed to a simulated viral infection by injection with the toll-like receptor 3 (TLR3) agonist poly(IC). This produces offspring with neurodevelopmental abnormalities associated with both autism spectrum disorders [59] and schizophrenia [60]. We administered ATP or saline to adult MIA offspring of poly(IC)-treated females and wild-type control offspring from saline-treated dams. We used a lower dose of 0.05 μmoles/g in females compared to 0.2 μmol/g in males because of the increased sensitivity of females to the hypothermic effects of ATP. All animals were 8–9 months of age. This is the human biological age equivalent of 35–38 years of age (see Materials and Methods). When the male MIA animals were given 0.2 μmol/g ATP, they had a 3.6 ± 0.3°C mean decrease in temperature (Fig 5A). MIA females had a 2.5 ± 0.3°C mean reduction in temperature following the 0.05 μmol/g dose of ATP (Fig 5B). Although MIA males (Fig 5A, gold triangles) started with a lower body temperature than controls (Fig 5A, red squares), the short-term hypothermic response to ATP injection measured over 1 hour was similar in magnitude and duration.

### The subacute temperature response to eATP

We next recorded the body temperatures in MIA animals over 5 days after a single injection of 0.2 μmoles/g ATP (Fig 5C and 5D). This experiment unmasked a sharp difference between control and MIA animals. ATP injection produced a significant rise in basal body temperature for days 1–3 after injection only in the MIA mice (gold triangles; 0.7 ± 0.1˚ in males, p < 0.0001; 0.6 ± 0.1˚ in females, p< 0.003), and not the unprimed wild-type controls (red squares, Fig 5C and 5D). MIA animals treated with saline remained at their baseline temperature and did not have an increase in temperature (Fig 5C and 5D, purple triangles). The core body temperature of the MIA mice then returned to baseline by 5 days after ATP injection (Fig 5C and 5D, gold triangles).

### The triphasic temperature response

We next followed the basal body temperature in 8-month old MIA males and controls for 28 days after a postnatal dose of poly(IC) or saline (Fig 5E). As first reported in 2013 [3], the MIA animals had a basal body temperature that was 0.5˚C lower than control animals (see Fig 5E, purple and gold MIA lines compared to the black and red control lines at time 0: control temperature = 36.3 ± 0.3˚C vs poly(IC) = 35.8 ± 0.4; 0.5˚C difference, p <0.03). Injection of poly(IC) unmasked a triphasic temperature response in both the MIA and control animals as follows: 1) An initial increase in temperature on day 1 after poly(IC) (red and gold squares), 2) a decrease in temperature on days 2–4 to below the pre-challenge baseline, 3) a return to baseline in control animals by 5 days (red squares), or a rebound increase of 0.8°C that was sustained between 6 to 14 days (36.3 ± 0.5 vs 35.5 ± 0.2; p <0.0001) in the MIA animals challenged with poly(IC), with a gradual return to baseline by 28 days (gold squares). Poly(IC) injection in both the MIA and control mice produced a similar magnitude of hypothermia on days 2 to 4 (Fig 5E, red and gold squares). These results revealed an unexpected rebound increase in body temperature during phase 3 of the triphasic response in MIA animals that did not occur in control animals given the same challenge.

## Discussion

The studies described in this paper are the first to use the newly available systems biology tools of metabolomics and breathomics, along with calorimetry, temperature, cytokine, hormone, and behavioral analysis to show that injection of the classical purinergic effector ATP had a profound, multisystem effect on both metabolism and behavior. Both short- and long-acting effects were found. The metabolism of purines, along with its connection to amino acid, methylation, glutathione, polyamine, urea cycle, and biopterin metabolism is illustrated in S9 Fig in S1 File. Extracellular ATP is rapidly metabolized by cell surface ectonucleotidases like CD73 and CD39 [61, 62] and other purine metabolic enzymes that include adenosine deaminase (ADA) and purine nucleoside phosphorylase (PNP) [63], AMP deaminase, IMP dehydrogenase, GMP synthase, guanine deaminase [33] and xanthine oxidase [64]. Metabolism of ATP leads to the production of ADP, AMP, and adenosine, which can each bind different purinergic receptors, transporters, and other proteins, and lead to hypometabolism and can cause hypothermia by several different mechanisms [65]. The role of adenine nucleotides and adenosine in lowering the metabolic rate in the context environmental stress [65–68], chronic pain [69], and non-REM sleep [70] has been studied for many years. Other purine metabolites like inosine, xanthosine, and xanthine are also produced by metabolic transformation of ATP. Xanthine is a source of reactive oxygen species (ROS) such as superoxide and hydrogen peroxide through the action of xanthine oxidase. Uric acid produced from xanthine is an activator of the NLRP3 inflammasome [71]. Even ATP itself, through its effect on P2X7 signaling, is a known activator of NLRP3 [72]. All the behavioral and the majority of metabolic effects of ATP injection reversed spontaneously within a few hours in unprimed typically developing animals. However, some metabolic effects did not emerge until 4 hours after injection and others occurred at 30 minutes and persisted for more than 4 hours. Other metabolic changes showed opposite directions of change at 30 minutes and 4 hours, consistent with an action-reaction, hormesis-like response [73–75]. In primed animals from the MIA model, eATP or poly(IC) exposure produced effects that lasted for days to weeks. The discovery of this latent memory response helps to understand how the cascade of mitochondrial and metabolic changes that occur acutely in response to stress or injury may initially be adaptive but can later lead to future hypersensitivities, developmental abnormalities, or lifelong health consequences [76].

Another major result of this study was that complex behaviors require a dialog between brain and body metabolism that is not controlled exclusively by neurotransmitters. The brain-body dialog is communicated along many channels—autonomic and neuroendocrine circuits, chemokines and cytokines from the immune system, reactive oxygen species (ROS), and metabolites and metabokines from all the organ systems including the microbiome. Small molecules produced locally then amplify and spread the signal using proteins, exosomes, microRNAs, and other molecules. The immune system can profoundly affect behavior even in the absence of infection. For example, interferon injection reproducibly triggers a physiologic and behavioral syndrome of fatigue, fever, headache, muscle aches and other symptoms such as social withdrawal that are indistinguishable from authentic viral infections [77]. This set of behaviors is evolutionarily conserved and known as stereotyped sickness behavior [78], which has been shown recently to be initiated by purinergic signaling [79].

We found that systemic ATP injection created a reversible hypometabolic state, dropping the whole-body metabolic rate by 74%. Since mitochondria are responsible for over 90% of whole-body oxygen consumption, the drop in oxygen consumption showed that systemic eATP was a potent inhibitor of whole-body mitochondrial aerobic metabolism. While this degree of inhibition of mitochondrial aerobic metabolism may at first seem maladaptive, it has been shown in many organisms exposed to environmental stress that transient induction of hypometabolic states such as dauer can confer a powerful survival advantage, but at the cost of a reversible decrease in maximum functional capacity [80]. The concept of reversibility is key. Short-term changes in mitochondrial function that are reversible can lead to long-term benefits over a lifetime. For example, in the context of microbial infection, reduction in mitochondrial bioenergetic capacity is part of the natural antiviral response [81].

The brain can be viewed as a state engine that simultaneously monitors information from the environment and the body using past experience to regulate the behavioral response to metabolism and optimize survival. Behavior and metabolism are inextricably intertwined. It is well-established that both genes and environment play a role in the genesis of autism spectrum disorder [82]. Metabolism in real time (M_t_) can be seen as the interaction of genes (G) and environment (E), conditioned by the timing of past environmental exposures during critical developmental windows, weighted (w*f(t)dt) and summed (Σ) over time (E_Σw*f(t)dt_), such that G x E_Σw*f(t)dt_ = M_t_. A more quantitative expression would need to include strongly interacting environmental factors that can occur within a discrete window of time of increased vulnerability, such as a second head trauma before the first is healed in sports [83], or sequential infectious, chemical, physical, or social stresses during early child development [13]. In all cases, a robust and evolutionarily tuned response to danger is paramount for survival and adaptation to both predictable and unpredictable changes in the environment throughout life.

The nature of chemical alarm signals used by plants and animals has been an active area of research since the classical papers by the Nobel-prize winning ethologist Karl von Frisch in the 1930s and 40s [84, 85] in which he coined the term *Schreckstoff*—the German word for fright or alarm substance. More recent studies have shown that Schreckstoff studied in fish contains purine metabolites [86] and glycosaminoglycans [87] that simultaneously serve as critical components of cellular defense, innate immunity and the inflammatory response to common environmental dangers like bacteria and water molds, and resistance to damaging effects of UV light, while also triggering stereotyped anxiety-associated behaviors [88, 89]. Plants also use ATP release to trigger reactive oxygen species and calcium in response to physical injury and stress [90]. In mice, the purine metabolite xanthine has recently been shown to trigger anxiety-associated behaviors, social abnormalities, and to facilitate both immunologic memory and stress-associated learning. This occurs via signaling pathways that begin with mitochondrial fragmentation and ultimately change behavioral responses via adenosine A1 receptor-mediated effects on oligodendroglia in the amygdala [91].

### Metabolomics

One of the most striking metabolic effects of ATP injection was the generalized decrease in all measured plasma amino acids within 30 minutes of the hyperpurinergic stimulus. The average decrease across 19 of 19 measured amino acids was -5.0 ± 2.6 standard deviations compared to saline controls. The pool of amino acids in the plasma is exchanged rapidly with tissues and is highly regulated [92]. It is currently unknown which tissues played the greatest role in regulating plasma amino acids in response to ATP signaling. If for example, the uptake is increased, or release is decreased from muscle, the resulting plasma depletion could trigger an amino acid depletion response in another tissue like liver. Such compartmentalization of the amino acid depletion response could lead to the accumulation of uncharged tRNAs and the activation of transcription factors like GCN2. GCN2 initiates a program of metabolic changes associated with the amino acid depletion response that stimulates autophagy and decreases inflammation and mammalian target of rapamycin complex 1 (mTORC1) signaling. Because protein synthesis imposes a significant energy cost on the cell, the amino acid response preserves ATP and GTP, and contributes to the hypometabolic response. GCN2 activation protects the cell from environmental danger [93, 94], and contributes to longevity [95], but at the cost of inhibited healing [96]. The role of extracellular ATP signaling in the GCN2-on/mTORC1-off mediated amino acid depletion response has not yet been investigated.

Vitamins are essential cofactors for enzymes that catalyze thousands of metabolic reactions in the cell. Without an adequate concentration of vitamins in cells, many metabolic reactions will slow or stop. Therefore, a concerted mechanism for rapidly changing the blood concentration of vitamins in response to environmental threat would have the effect of rapidly changing the flux of metabolites through all the biochemical pathways controlled by vitamin availability. Mitochondria are especially rich in enzymes requiring the B vitamins. Plasma levels of thiamine (B1), niacin (B3), pyridoxic acid (B6), FAD (B2), methyl-tetrahydrofolic acid (mTHF, B9), and carnitine were each increased in the plasma after ATP injection. This implies that the levels in mitochondria in at least some tissue was decreased. Reciprocally, the plasma levels of the vitamin D precursor cholecalciferol (vitamin D3) were rapidly decreased by ATP injection. This implies that the cellular levels of vitamin D3 in at least some tissues were correspondingly increased. These examples illustrate that the mobilization of vitamins and conditionally-essential nutrients like carnitine during stress may deplete intracellular pools in some organs in exchange for making them available in the plasma for other tissue types. What about the chronic effects? If, for example a significant amount of FAD and niacin is released by liver cells into the plasma and this is sustained for more than a few hours, then mitochondrial fatty acid oxidation in the liver, which like all tissues requires FAD and NAD+ from niacin as cofactors, might be decreased and lead to abnormalities in plasma acyl-carnitines. Abnormalities in fatty acid oxidation and acyl-carnitine profiles in children with ASD are well known [97]. Our results showed that short and medium chain acyl-carnitines like acetyl- (C2), propionyl- (C3), and hexanoyl- (C6) carnitine were decreased, while medium chain dicarboxylic acid acyl-carnitines like glutaryl- (C5DC) and adipoyl- (C6DC) carnitine were increased. The effect of purinergic signaling on the redistribution of intracellular vitamin pools in different organs like the liver or muscle, and on excretion in the urine has not yet been investigated. Longer-term elevation in the plasma levels of FAD and carnitine could lead to greater urinary filtration and greater excretion that could lower total body pools and produce chronic effects on mitochondrial fatty acid metabolism. Over time, whole-body carnitine deficiency could result that is not caused by deficient intake, but rather by excess excretion. Low carnitine will lead to abnormalities in long-chain acyl-carnitines because carnitine esterification is needed for normal transport and oxidization of long-chain fatty acids in mitochondria. Likewise, if elevated levels of 5’-methyltetrahydrofolate (mTHF) persist after a pulse of purinergic signaling, increased urinary excretion might lower total body pools of mTHF despite adequate intake and produce reductions in methylation needed for nucleic acid and neurotransmitter synthesis, and methyl-B12 metabolism, which are each known issues in children with ASD [97–99]. The long-term metabolomic effects of eATP exposure were not examined in these studies.

Another surprising effect of ATP injection was the dramatic increase in metabolites known to be derived predominantly or entirely from microbial metabolism in the gut. Thirteen of 20 measured microbiome metabolites were changed and 11 of these were increased 30 minutes after ATP injection. We hypothesize that systemic exposure to ATP and ADP may increase intestinal permeability by regulating zonulin located in the tight junctions between intestinal epithelial cells and lead to the phenomenon known as a leaky gut [100]. One caveat to this interpretation is that the purinergic stimulus in this study was given by i.p. injection, which transiently exposes the serosal surface of the intestines to higher concentrations of eATP before it is absorbed into the blood. This may amplify the effect of eATP on tight junctions. On the other hand, it has been shown that functional changes in mitochondria directly change the microbiome [24] and intestinal epithelial cell and mucosal function at the host-microbe interface [101]. The inhibition of mitochondrial function by systemic eATP shown in this study might therefore be a direct cause of intestinal epithelial cell functional changes that lead to leaky gut and chronic changes in the microbial ecology. The differential effects of i.p. vs i.v. ATP injection on microbiome metabolites and more direct measures of leaky gut have not yet been studied.

### Breathomics

Breathomics analysis showed that ATP injection stimulated an increase in several small volatile organic compounds (VOCs) from 1 to 5 carbons in length. The increase in one-carbon redox series carbon monoxide (CO), methanol, and methane supports the concept of a relative block in macromolecular polymer synthesis in the form of new proteins, lipids, nucleic acids, and polysaccharides. CO is produced by heme oxygenase I under conditions of cell stress and is a potent inhibitor of mitochondrial cytochrome c oxidase [102]. Dimethylsulfide (DMS) was also increased. Recent work in patients with diabetes has shown a strong correlation between exhaled DMS and hydrogen sulfide (H_2_S) in the blood [103]. Both CO and H_2_S are potent inhibitors of mitochondrial oxygen consumption [104]. Because oxygen extraction from the blood is decreased when mitochondrial cytochrome c oxidase in inhibited, intracellular levels of dissolved oxygen can rise in response to physiologic tissue levels of CO and H_2_S. This can make additional dissolved oxygen available for ROS production by cell defense molecules like NADPH oxidase [105]. The shift toward oxidizing conditions minimizes the chances that an invading microbe can usurp cellular resources for synthesizing its own polymers, and leads naturally to the accumulation of small molecular weight intermediates and monomers [17].

Isoprene is a 5-carbon, branched chain, volatile liquid that was also elevated after ATP injection. Isoprene is the most abundant VOC and accounts for 70% of all hydrocarbons found in human breath [106]. Isoprene is made from a building block of cholesterol called isopentenyl pyrophosphate (IPP) and its isomer dimethylallyl pyrophosphate (DMAPP) [107] (S12 Fig in S1 File). When more isoprene is released, less IPP and DMAPP are available for downstream synthesis not only of cholesterol, but also other isoprenoids like cortisol, steroid hormones, bile acids, dolichols for glycoprotein synthesis, the electron carrier CoQ10, isopentenyl-modified tRNAs for selenoprotein synthesis, and prenylation of key signaling proteins like Ras and Rho [108, 109]. IPP is increased when the genes for the enzymes that process it like isopentenyl diphosphate isomerase 1 and 2 (IDI1 and IDI2) are disrupted in a copy number variant in amyotrophic lateral sclerosis [110], or when the protein is aggregated in Lewy bodies in the brain [111]. Rapamycin has been shown to inhibit stem cell growth by indirectly inhibiting IDI and creating a metabolic bottleneck in isoprenoid synthesis. Upstream of the bottleneck IPP is increased, while downstream isoprenoids needed for cell growth are depleted. Replacement of products downstream of the IDI bottleneck like DMAPP or farnesyl pyrophosphate reversed the growth inhibitory effects of rapamycin [112]. Rapamycin is more classically known to inhibit the insulin-like growth factor receptor (IGF1R), phosphatidylinositol-3-kinase (PI3K), AKT, mammalian target of rapamycin (mTOR) (PI3/AKT/mTOR) signaling pathway by binding to mTOR, but the metabolic mechanism for the resulting cell growth inhibition remained unknown until these recent studies. The PI3/AKT/mTOR pathway and its regulation by the protein known as the phosphatase and tensin homolog (PTEN), have long been known to be important players in the pathogenesis of autism [113]. IPP is a potent activator of γδ-T cells that facilitates antimicrobial and anticancer cell killing [114]. IPP is also needed for the tRNA modification needed to synthesize over two dozen stress-related selenoproteins like the glutathione peroxidases and thyroxine deiodinases [115] (S12 Fig in S1 File). Isoprene in the breath has been measured in children and found to be increased with steroid hormone synthesis in puberty [116], and in adults after exercise and even with the normal orthostatic stress of standing up after sitting or reclining [117]. These recent findings help to place IPP and exhaled isoprene into the context of ASD and the normal stress of development, exercise, inflammation, immunity, and the cell danger response.

### The MIA eATP and poly(IC) temperature responses, ME/CFS, and ASD

We found that the baseline temperature of MIA animals was 0.5˚C (0.9˚F) lower than controls. This has been reported previously in MIA mice [3] and was similar to the chronic reduction in basal body temperature that is seen in some children with autism [118]. These findings were consistent with the hypothesis that mild reduction in body temperature might be a biomarker of a primed and persistently activated state of the cell danger response (CDR). In the MIA model this was produced by maternal exposure to the poly(IC) as the CDR trigger. In children with ASD, the persistent CDR can be caused by a large number of genetic and environmental factors that may all share purinergic signaling as a common denominator. Previous studies have shown that treatment of the MIA animals with the antipurinergic drug suramin completely restored normal body temperature at the same time as restoring normal social behaviors [3]. While MIA and control animals had a similar hypothermic response to eATP challenge in the first hour, they differed when their physiologic responses were measured over the following days and weeks. When challenged with eATP MIA animals showed a rebound increase in temperature that started 1 day after the challenge and lasted for 3–5 days. This pattern was similar to the phenomenon known as post-exertional malaise (PEM) in patients with ME/CFS. In ME/CFS, PEM can be triggered by either physical, cognitive or emotional stress [119]. Each of these stresses can be shown to result in increased eATP release from cells or synapses.

The subacute eATP effect may be relevant to the fever response in children with ASD. Many children with ASD experience a short-term improvement in the core symptoms of ASD during fever [120]. Once the acute infection subsides and the temperature falls, the symptoms of ASD return [120]. We did not test the ASD-related behaviors like social approach in the MIA animals in the hours and days following postnatal challenge with eATP when their temperature remained increased. In contrast to eATP, poly(IC) is multifaceted trigger of the cell danger response that simulates an RNA virus infection. In addition to eATP release from exposed cells, poly(IC) is a ligand for TLR3 signaling. This one-two punch of poly(IC) produced a rebound increase in temperature in MIA animals that lasted for nearly 4 weeks. Control animals returned to normal after the poly(IC) challenge in just 3–5 days. MIA animals were not studied in the CLAMS cages, so the bioenergetic effects associated with the rebound temperature response are not known. Future studies will be needed to quantify the ASD-like behaviors during each phase of the subacute and triphasic temperature response to generic innate immune triggers like ATP and poly(IC) to test the similarity to PEM in human ME/CFS, and the ASD fever response.

### Purinergic signaling as a common denominator

Our results support the emerging conclusion that abnormalities in purinergic signaling may be a common denominator for many different neurodevelopmental [121], affective, neuropsychiatric, and neurodegenerative disorders [122, 123]. Abnormalities in purinergic signaling persist when cells fail to resolve the cell danger response (CDR). This is associated with checkpoint blocks in the healing cycle. The healing cycle is a highly regulated, choreographed, and evolutionarily conserved sequence of metabolic and behavioral events initiated in response to stress or injury [124]. Our results support the hypothesis that it is not the genetic or environmental stress per se, but rather the *biological response* to the stress by the brain-body system that causes both adaptive and maladaptive behaviors, and the physiologic changes that underlie the signs and symptoms of a chronic disorder. New antipurinergic therapies (APTs) with suramin-like actions designed to rebalance cell danger signaling and remove blocks to the healing cycle might prove therapeutic in clinical conditions characterized by persistent activation of the cell danger response such as autism [1, 2, 4], myalgic encephalomyelitis/chronic fatigue syndrome (ME/CFS) [125], Gulf War illness [126], post-traumatic stress disorder (PTSD), major depressive disorder (MDD) and bipolar disorder (BD) [127], amyotrophic lateral sclerosis (ALS) [128], and several other developmental, neurodegenerative, and age-related disorders [17, 96].

In children and adults with ASD, the hypersensitivity to purinergic agonists and to other innate immune triggers like infections that inevitably trigger ATP release, would mean that relatively small environmental stresses can cause exaggerated and prolonged metabolic and behavioral responses. Under these circumstances of increased sensitivity to eATP signaling, the brain may produce dramatically different behaviors in response to the same metabolic signals. For example, in typically developing children, a given amount of ATP released might produce no observable response or a blunted response that returns quickly to baseline. While in a child with ASD, the same pulse of eATP could produce hyperreactive behaviors and cascading metabolic responses that might last for hours to weeks, disrupt development, and oppose the beneficial effects of ongoing supplement, drug, and behavioral therapies. Repeated pulses of eATP signaling in the brain, gastrointestinal, and immune cells of children with ASD could provide a unifying mechanism for the observed mitochondrial dysfunction [129], acyl-carnitine abnormalities [97], leaky gut [130], reactive oxygen and microbiome abnormalities [24, 131], mast cell abnormalities [132, 133], dysregulated innate and adaptive immune system signaling [134], activated microglia [135, 136], reduced synaptic plasticity [137], altered calcium homeostasis [138, 139], lowered seizure threshold [140], brain growth abnormalities [141, 142], and altered child neurodevelopmental trajectories [143]. Several endophenotypes of autism are known [144] that might be explained by a predominance of one or more of the above symptoms that each trace back to eATP signaling and mitochondria. The results of this study support the hypothesis that once initiated by eATP, the signs and symptoms that make each child with ASD unique are determined by a personalized combination of risk and resilience genes that are expressed early, and environmental exposures that have occurred during critical developmental windows.

Additional evidence for purinergic signaling as a final common denominator in ASD models comes from the work of Horvath, et al. [145]. In this paper it was shown that injection of ATP itself into pregnant females was sufficient to produce the post-natal cerebellar Purkinje cell dropout and life-long autism-like behaviors that were indistinguishable from the classical MIA model. Gestational poly(IC) exposure was not necessary. Extracellular ATP and its metabolite ADP are released from cells as danger signals in response to nearly every physical, microbial, inflammatory, chemical, or metabolic stress studied to date [11, 15, 16, 146]. Because mitochondria serve as the substrate for metabolic memory [147], eATP effects on mitochondria will naturally affect metabolic memory. Mitochondrial adaptations to past stresses and quality control are crucial in regulating metabolism, innate immunity, cellular defense [148], neurodevelopment, and behavior from conception to old age [76]. The effect of eATP on mitochondrial metabolism is necessary to initiate both inflammation and the healing cycle. When healing is complete, extracellular ATP levels decrease because healthy cells metabolize more eATP than they release. Once normal purinergic signaling is restored, the normal functions of mitochondria shift away from cellular defense and back to normal functions needed for child development and healthy aging [124]. We hypothesize that when the molecular steps of the healing cycle do not resolve spontaneously after a stress has passed, the cell danger response is maintained by persistent purinergic signaling. When abnormalities in purinergic signaling occur during critical windows for child and young adult development chronic illness or disability may result [124].

### Limitations

Only one strain of mouse, the C57Bl/6J strain, was used in this study. Although this is the classic laboratory mouse strain used in the MIA model, other genetic backgrounds such as the FVB mouse used in our previous studies of Fragile X syndrome [1], could theoretically show different metabolic responses to ATP injection. However, the specific genetic differences between mouse strains, and even specific mutations leading to ASD, appear not to have a significant effect on purinergic signaling associated with ASD-like behaviors. Several groups have now shown that treatment with the antipurinergic drug suramin was able to correct all the behavioral abnormalities, and most of the metabolic abnormalities, in the MIA model in C57Bl/6J mice, the Fragile X model in FVB mice [1–3], a rat model of ASD caused by prenatal exposure to valproic acid [149], and in a small clinical trial in children with ASD [4]. Metabolomic analysis after correction of the ASD-associated behaviors showed that the top metabolic pathway changed by treatment in all of these studies was purines [1, 2, 4]. ATP injection in rats is also known to cause hypothermia and pro-inflammatory effects in the brain [150] but metabolomics has not yet been performed. Intravenous infusions of ATP have been reported in patients with advanced cancer [151]. However, none of the published studies in humans have reported body temperature or metabolomic responses before and after the i.v. infusion. Another limitation is that the synergistic effects of the autonomic nervous system and neuroendocrine systems triggered by purinergic signaling, beyond the acute cortisol response, were not examined in this study. However, based on the known effects of reactive oxygen species, ATP, and other purinergic effectors on the microbiome [24], vagus nerve [152], adrenal function [153], and even hypothalamic function [154] via circumventricular organs that lack a blood brain barrier [155], these synergistic multi-system effects are likely to be significant. The generalizability of the current mouse studies to larger studies in children with ASD is unknown.

## Conclusions

The effects of extracellular ATP were pleiotropic. ATP injection produced dramatic changes in behavior that were similar to anxiety-associated behaviors that have been studied in many species and associated with alarm signaling [88, 91]. Some of the behaviors produced by eATP were similar to those quantified previously in the MIA model of ASD, such as open field avoidance and gait coordination abnormalities [3, 156]. A major difference was that these behaviors were transient after ATP injection and returned to normal as metabolism and mitochondrial function returned to normal in a few hours. Metabolic and mitochondrial changes were inextricably tied to the behavioral responses. Metabolic changes included decreased whole body oxygen consumption, metabolic rate and temperature. Mitochondrial changes were documented by changes in amino acid, fatty acid, nucleotide, phospholipid, bile acid, redox, vitamin, microbiome, and energy metabolism. Many changes were consistent with rapid alterations in the compartmental distribution of metabolites between plasma and cells.

We found that males were more sensitive to the *behavioral* effects of systemic eATP. Females were more sensitive to the *metabolic* effects of eATP. Female mice show fewer ASD-associated behaviors compared to their male littermates in the MIA mouse model [3]. The greater sensitivity of females to the metabolic effects of ATP signaling may contribute to the 3 to 1 increased risk of females compared to males for another complex disorder, myalgic encephalomyelitis/chronic fatigue syndrome (ME/CFS) [157]. The increased behavioral response in males to ATP signaling and decreased metabolic response may contribute to the 4 to 1 increased risk of males compared to females for the development of ASD. MIA animals were hypersensitive to postnatal exposures to CDR triggers like poly(IC) and extracellular ATP (eATP) even as adults. The hypersensitivity to extracellular purines and poly(IC) in the MIA model may be relevant for pulsed or persistent activation of the CDR by environmental stresses of many kinds known to change child development, including early life stress [158] and environmental pollution [20, 159].

## Supporting information

S1 File (PDF)

S1 Table Raw metabolomics AUC data. (XLSX)

S2 Table Statistical analysis of metabolomics 30 minutes post-ATP injection. (XLSX)

S3 Table Rank order of biochemical pathways changed at 30 minutes. (XLSX)

S4 Table Rank order of metabolite clusters by k-NN analysis—30 minutes after ATP injection. (XLSX)

S5 Table Top k-NN cluster of metabolites increased at 30 minutes. (XLSX)

S6 Table Top k-NN cluster of metabolites decreased at 30 minutes. (XLSX)

S7 Table Statistical analysis of metabolomics 4 hours post-ATP injection. (XLSX)

S8 Table Rank order of biochemical pathways changed at 4 hours. (XLSX)

S9 Table Rank order of metabolite clusters by k-NN analysis—4 hours after ATP injection. (XLSX)

S10 Table Top k-NN cluster of metabolites increased at 4 hours. (XLSX)

S11 Table Top k-NN clusters of metabolites decreased at 4 hours. (XLSX)

S12 Table Ranking of ATP-responsive metabolites by 3-group ANOVA. Saline, 30 min, and 4 h.
(XLSX)

## References

[1] Naviaux JC, Wang L, Li K, Bright AT, Alaynick WA, Williams KR, et al. Antipurinergic therapy corrects the autism-like features in the Fragile X (Fmr1 knockout) mouse model. Molecular autism. 2015;6:1. 10.1186/2040-2392-6-1 25705365 PMC4334917

[2] Naviaux JC, Schuchbauer MA, Li K, Wang L, Risbrough VB, Powell SB, et al. Reversal of autism-like behaviors and metabolism in adult mice with single-dose antipurinergic therapy. Translational psychiatry. 2014;4:e400. 10.1038/tp.2014.33 . 24937094 PMC4080315

[3] Naviaux RK, Zolkipli-Cunningham Z, Nakayama T, Naviaux JC, Le T, Wang L, et al. Antipurinergic Therapy Corrects the Autism-Like Features in the Poly(IC) Mouse Model. PloS one. 2013;8(3):e57380. 10.1371/journal.pone.0057380 23516405 PMC3596371

[4] Naviaux RK, Curtis B, Li K, Naviaux JC, Bright AT, Reiner GE, et al. Low-dose suramin in autism spectrum disorder: a small, phase I/II, randomized clinical trial. Ann Clin Transl Neurol. 2017;4(7):491–505. 10.1002/acn3.424 28695149 PMC5497533

[5] Burnstock G, Campbell G, Satchell D, Smythe A. Evidence that adenosine triphosphate or a related nucleotide is the transmitter substance released by non-adrenergic inhibitory nerves in the gut. British journal of pharmacology. 1970;40(4):668–88. Epub 1970/12/01. 10.1111/j.1476-5381.1970.tb10646.x . 4322041 PMC1702901

[6] Burnstock G. Purine and purinergic receptors. Brain Neurosci Adv. 2018;2:2398212818817494. Epub 2018/12/06. 10.1177/2398212818817494 32166165 PMC7058212

[7] Ledderose C, Liu K, Kondo Y, Slubowski CJ, Dertnig T, Denicolo S, et al. Purinergic P2X4 receptors and mitochondrial ATP production regulate T cell migration. The Journal of clinical investigation. 2018;128(8):3583–94. Epub 2018/06/13. 10.1172/JCI120972 29894310 PMC6063471

[8] Ledderose C, Bromberger S, Slubowski CJ, Sueyoshi K, Junger WG. Frontline Science: P2Y11 receptors support T cell activation by directing mitochondrial trafficking to the immune synapse. J Leukoc Biol. 2020. Epub 2020/06/13. 10.1002/JLB.2HI0520-191R . 32531829 PMC8772287

[9] Burnstock G. Purinergic cotransmission. Experimental physiology. 2009;94(1):20–4. Epub 2008/08/30. 10.1113/expphysiol.2008.043620 . 18723580

[10] Lockhart RA, Spinelli JJ, Stephens MA. Cramér–von Mises statistics for discrete distributions with unknown parameters. The Canadian Journal of Statistics/La Revue Canadienne de Statistique. 2007:125–33.

[11] Burnstock G, Knight GE. Cell culture: complications due to mechanical release of ATP and activation of purinoceptors. Cell Tissue Res. 2017. 10.1007/s00441-017-2618-8 . 28434079 PMC5610203

[12] Sakaki H, Tsukimoto M, Harada H, Moriyama Y, Kojima S. Autocrine regulation of macrophage activation via exocytosis of ATP and activation of P2Y11 receptor. PloS one. 2013;8(4):e59778. Epub 2013/04/12. 10.1371/journal.pone.0059778 23577075 PMC3618444

[13] Mackes NK, Golm D, Sarkar S, Kumsta R, Rutter M, Fairchild G, et al. Early childhood deprivation is associated with alterations in adult brain structure despite subsequent environmental enrichment. Proceedings of the National Academy of Sciences of the United States of America. 2020;117(1):641–9. Epub 2020/01/08. 10.1073/pnas.1911264116 31907309 PMC6955353

[14] Michaletti A, Gioia M, Tarantino U, Zolla L. Effects of microgravity on osteoblast mitochondria: a proteomic and metabolomics profile. Sci Rep. 2017;7(1):15376. Epub 2017/11/15. 10.1038/s41598-017-15612-1 29133864 PMC5684136

[15] Heil M, Land WG. Danger signals—damaged-self recognition across the tree of life. Front Plant Sci. 2014;5:578. 10.3389/fpls.2014.00578 25400647 PMC4215617

[16] Pittman K, Kubes P. Damage-associated molecular patterns control neutrophil recruitment. Journal of innate immunity. 2013;5(4):315–23. 10.1159/000347132 . 23486162 PMC6741494

[17] Naviaux RK. Metabolic features of the cell danger response. Mitochondrion. 2014;16:7–17. 10.1016/j.mito.2013.08.006 . 23981537

[18] Wallace DC. Bioenergetics in human evolution and disease: implications for the origins of biological complexity and the missing genetic variation of common diseases. Philosophical transactions of the Royal Society of London Series B, Biological sciences. 2013;368(1622):20120267. Epub 2013/06/12. 10.1098/rstb.2012.0267 23754818 PMC3685467

[19] Wallace DC, Fan W. Energetics, epigenetics, mitochondrial genetics. Mitochondrion. 2010;10(1):12–31. 10.1016/j.mito.2009.09.006 19796712 PMC3245717

[20] Naviaux RK. Perspective: Cell danger response biology-The new science that connects environmental health with mitochondria and the rising tide of chronic illness. Mitochondrion. 2020;51:40–5. Epub 2019/12/27. 10.1016/j.mito.2019.12.005 . 31877376

[21] Naviaux RK, Le TP, Bedelbaeva K, Leferovich J, Gourevitch D, Sachadyn P, et al. Retained features of embryonic metabolism in the adult MRL mouse. Molecular genetics and metabolism. 2009;96(3):133–44. 10.1016/j.ymgme.2008.11.164 19131261 PMC3646557

[22] Ginsberg MR, Rubin RA, Falcone T, Ting AH, Natowicz MR. Brain transcriptional and epigenetic associations with autism. PloS one. 2012;7(9):e44736. 10.1371/journal.pone.0044736 22984548 PMC3440365

[23] Gevi F, Zolla L, Gabriele S, Persico AM. Urinary metabolomics of young Italian autistic children supports abnormal tryptophan and purine metabolism. Molecular autism. 2016;7:47. Epub 2016/12/03. 10.1186/s13229-016-0109-5 27904735 PMC5121959

[24] Yardeni T, Tanes CE, Bittinger K, Mattei LM, Schaefer PM, Singh LN, et al. Host mitochondria influence gut microbiome diversity: A role for ROS. Sci Signal. 2019;12(588). Epub 2019/07/04. 10.1126/scisignal.aaw3159 . 31266851

[25] Howsmon DP, Vargason T, Rubin RA, Delhey L, Tippett M, Rose S, et al. Multivariate techniques enable a biochemical classification of children with autism spectrum disorder versus typically-developing peers: A comparison and validation study. Bioeng Transl Med. 2018;3(2):156–65. Epub 2018/08/02. 10.1002/btm2.10095 30065970 PMC6063877

[26] Howsmon DP, Kruger U, Melnyk S, James SJ, Hahn J. Classification and adaptive behavior prediction of children with autism spectrum disorder based upon multivariate data analysis of markers of oxidative stress and DNA methylation. PLoS computational biology. 2017;13(3):e1005385. Epub 2017/03/17. 10.1371/journal.pcbi.1005385 28301476 PMC5354243

[27] Nyhan WL, James JA, Teberg AJ, Sweetman L, Nelson LG. A new disorder of purine metabolism with behavioral manifestations. The Journal of pediatrics. 1969;74(1):20–7. Epub 1969/01/01. 10.1016/s0022-3476(69)80004-1 . 5782823

[28] Becker MA, Smith PR, Taylor W, Mustafi R, Switzer RL. The genetic and functional basis of purine nucleotide feedback-resistant phosphoribosylpyrophosphate synthetase superactivity. The Journal of clinical investigation. 1995;96(5):2133–41. Epub 1995/11/01. 10.1172/JCI118267 7593598 PMC185862

[29] Page T, Coleman M. Purine metabolism abnormalities in a hyperuricosuric subclass of autism. Biochimica et biophysica acta. 2000;1500(3):291–6. Epub 2000/03/04. 10.1016/s0925-4439(99)00113-1 . 10699370

[30] Coleman M, Blass JP. Autism and lactic acidosis. Journal of autism and developmental disorders. 1985;15(1):1–8. Epub 1985/03/01. 10.1007/BF01837894 . 3980425

[31] Weissman JR, Kelley RI, Bauman ML, Cohen BH, Murray KF, Mitchell RL, et al. Mitochondrial disease in autism spectrum disorder patients: a cohort analysis. PloS one. 2008;3(11):e3815. Epub 2008/12/02. 10.1371/journal.pone.0003815 19043581 PMC2584230

[32] Rossignol DA, Frye RE. Mitochondrial dysfunction in autism spectrum disorders: a systematic review and meta-analysis. Molecular psychiatry. 2012;17(3):290–314. 10.1038/mp.2010.136 21263444 PMC3285768

[33] Micheli V, Camici M, Tozzi MG, Ipata PL, Sestini S, Bertelli M, et al. Neurological disorders of purine and pyrimidine metabolism. Curr Top Med Chem. 2011;11(8):923–47. 10.2174/156802611795347645 . 21401501

[34] Graf WD, Marin-Garcia J, Gao HG, Pizzo S, Naviaux RK, Markusic D, et al. Autism associated with the mitochondrial DNA G8363A transfer RNA(Lys) mutation. Journal of child neurology. 2000;15(6):357–61. 10.1177/088307380001500601 . 10868777

[35] Licznerski P, Park HA, Rolyan H, Chen R, Mnatsakanyan N, Miranda P, et al. ATP Synthase c-Subunit Leak Causes Aberrant Cellular Metabolism in Fragile X Syndrome. Cell. 2020;182(5):1170–85 e9. Epub 2020/08/17. 10.1016/j.cell.2020.07.008 32795412 PMC7484101

[36] Estes ML, McAllister AK. Maternal immune activation: Implications for neuropsychiatric disorders. Science. 2016;353(6301):772–7. Epub 2016/08/20. 10.1126/science.aag3194 27540164 PMC5650490

[37] Minakova E, Warner BB. Maternal immune activation, central nervous system development and behavioral phenotypes. Birth Defects Res. 2018;110(20):1539–50. Epub 2018/11/16. 10.1002/bdr2.1416 . 30430765

[38] Solek CM, Farooqi N, Verly M, Lim TK, Ruthazer ES. Maternal immune activation in neurodevelopmental disorders. Dev Dyn. 2018;247(4):588–619. Epub 2017/12/12. 10.1002/dvdy.24612 . 29226543

[39] Overton JM. Phenotyping small animals as models for the human metabolic syndrome: thermoneutrality matters. Int J Obes (Lond). 2010;34 Suppl 2:S53–8. 10.1038/ijo.2010.240 . 21151148

[40] Flurkey K, Currer J.M., Harrison D.E. Mouse Models in Aging Research. In: Fox J. G. ea, editor. The Mouse in Biomedical Research, 2nd edition. 3. San Diego, CA: Academic Press; 2007. p. 637–72.

[41] Golde WT, Gollobin P, Rodriguez LL. A rapid, simple, and humane method for submandibular bleeding of mice using a lancet. Lab animal. 2005;34(9):39–43. 10.1038/laban1005-39 . 16195737

[42] Li K, Naviaux JC, Bright AT, Wang L, Naviaux RK. A robust, single-injection method for targeted, broad-spectrum plasma metabolomics. Metabolomics: Official journal of the Metabolomic Society. 2017;13(10):122. 10.1007/s11306-017-1264-1 28943831 PMC5583274

[43] Langeroudi AG, Hirsch CM, Estabragh AS, Meinardi S, Blake DR, Barbour AG. Elevated carbon monoxide to carbon dioxide ratio in the exhaled breath of mice treated with a single dose of lipopolysaccharide. Open Forum Infect Dis. 2014;1(2):ofu085. Epub 2015/03/04. 10.1093/ofid/ofu085 25734151 PMC4281777

[44] Lin HY, Weng SW, Shen FC, Chang YH, Lian WS, Hsieh CH, et al. Abrogation of Toll-Like Receptor 4 Mitigates Obesity-Induced Oxidative Stress, Proinflammation, and Insulin Resistance Through Metabolic Reprogramming of Mitochondria in Adipose Tissue. Antioxidants & redox signaling. 2020;33(2):66–86. Epub 2020/01/18. 10.1089/ars.2019.7737 . 31950846

[45] Conti B, Sanchez-Alavez M, Winsky-Sommerer R, Morale MC, Lucero J, Brownell S, et al. Transgenic mice with a reduced core body temperature have an increased life span. Science. 2006;314(5800):825–8. Epub 2006/11/04. 10.1126/science.1132191 . 17082459

[46] Xia J, Sinelnikov IV, Han B, Wishart DS. MetaboAnalyst 3.0-making metabolomics more meaningful. Nucleic acids research. 2015;43(W1):W251–W7. 10.1093/nar/gkv380 . 25897128 PMC4489235

[47] Chong J, Soufan O, Li C, Caraus I, Li S, Bourque G, et al. MetaboAnalyst 4.0: towards more transparent and integrative metabolomics analysis. Nucleic acids research. 2018. Epub 2018/05/16. 10.1093/nar/gky310 . 29762782 PMC6030889

[48] Benjamini Y, Hochberg Y. Controlling the false discovery rate—a practical and powerful approach to multiple testing. Journal of the Royal Statistical Society Series B-Methodological. 1995;57(1):289–300. WOS:A1995QE45300017.

[49] Storey JD. The positive false discovery rate: a Bayesian interpretation and the q-value. The Annals of Statistics. 2003;31(6):2013–35.

[50] Breiman L. Random Forests. Machine Learning. 2001;45(1):5–32.

[51] Altman NS. An introduction to kernel and nearest-neighbor nonparametric regression. The American Statistician. 1992;46(3):175–85.

[52] Howes OD, McCutcheon R, Owen MJ, Murray RM. The Role of Genes, Stress, and Dopamine in the Development of Schizophrenia. Biol Psychiatry. 2017;81(1):9–20. Epub 2016/10/11. 10.1016/j.biopsych.2016.07.014 27720198 PMC5675052

[53] Kratzer JT, Lanaspa MA, Murphy MN, Cicerchi C, Graves CL, Tipton PA, et al. Evolutionary history and metabolic insights of ancient mammalian uricases. Proceedings of the National Academy of Sciences of the United States of America. 2014;111(10):3763–8. 10.1073/pnas.1320393111 24550457 PMC3956161

[54] Ghimire S, Matos C, Caioni M, Weber D, Peter K, Holler E, et al. Indoxyl 3-sulfate inhibits maturation and activation of human monocyte-derived dendritic cells. Immunobiology. 2018;223(2):239–45. Epub 2017/11/05. 10.1016/j.imbio.2017.10.014 . 29100619

[55] Kaneko K, Aoyagi Y, Fukuuchi T, Inazawa K, Yamaoka N. Total purine and purine base content of common foodstuffs for facilitating nutritional therapy for gout and hyperuricemia. Biol Pharm Bull. 2014;37(5):709–21. Epub 2014/02/21. 10.1248/bpb.b13-00967 . 24553148

[56] Ley K. Arrest chemokines. Microcirculation. 2003;10(3–4):289–95. Epub 2003/07/10. 10.1038/sj.mn.7800194 . 12851646

[57] Seo DR, Kim SY, Kim KY, Lee HG, Moon JH, Lee JS, et al. Cross talk between P2 purinergic receptors modulates extracellular ATP-mediated interleukin-10 production in rat microglial cells. Exp Mol Med. 2008;40(1):19–26. Epub 2008/02/29. 10.3858/emm.2008.40.1.19 18305394 PMC2679320

[58] Nishi H, Arai H, Momiyama T. NCI-H295R, a human adrenal cortex-derived cell line, expresses purinergic receptors linked to Ca(2)(+)-mobilization/influx and cortisol secretion. PloS one. 2013;8(8):e71022. 10.1371/journal.pone.0071022 23951072 PMC3738630

[59] Patterson PH. Modeling autistic features in animals. Pediatric research. 2011;69(5 Pt 2):34R–40R. Epub 2011/02/04. 10.1203/PDR.0b013e318212b80f 21289542 PMC3088489

[60] Bitanihirwe BK, Peleg-Raibstein D, Mouttet F, Feldon J, Meyer U. Late prenatal immune activation in mice leads to behavioral and neurochemical abnormalities relevant to the negative symptoms of schizophrenia. Neuropsychopharmacology: official publication of the American College of Neuropsychopharmacology. 2010;35(12):2462–78. Epub 2010/08/26. 10.1038/npp.2010.129 20736993 PMC3055332

[61] Allard B, Longhi MS, Robson SC, Stagg J. The ectonucleotidases CD39 and CD73: Novel checkpoint inhibitor targets. Immunological reviews. 2017;276(1):121–44. 10.1111/imr.12528 28258700 PMC5338647

[62] Antonioli L, Pacher P, Vizi ES, Hasko G. CD39 and CD73 in immunity and inflammation. Trends in molecular medicine. 2013;19(6):355–67. 10.1016/j.molmed.2013.03.005 23601906 PMC3674206

[63] Grunebaum E, Cohen A, Roifman CM. Recent advances in understanding and managing adenosine deaminase and purine nucleoside phosphorylase deficiencies. Curr Opin Allergy Clin Immunol. 2013;13(6):630–8. Epub 2013/10/12. 10.1097/ACI.0000000000000006 . 24113229

[64] Kushiyama A, Nakatsu Y, Matsunaga Y, Yamamotoya T, Mori K, Ueda K, et al. Role of Uric Acid Metabolism-Related Inflammation in the Pathogenesis of Metabolic Syndrome Components Such as Atherosclerosis and Nonalcoholic Steatohepatitis. Mediators of inflammation. 2016;2016:8603164. Epub 2017/01/11. 10.1155/2016/8603164 28070145 PMC5192336

[65] Carlin JL, Jain S, Gizewski E, Wan TC, Tosh DK, Xiao C, et al. Hypothermia in mouse is caused by adenosine A1 and A3 receptor agonists and AMP via three distinct mechanisms. Neuropharmacology. 2017;114:101–13. Epub 2016/12/05. 10.1016/j.neuropharm.2016.11.026 27914963 PMC5183552

[66] Zhao Z, Van Oort A, Tao Z, O’Brien WG 3rd, Lee CC. Metabolite Profiling of 5’-AMP-Induced Hypometabolism. Metabolomics: Official journal of the Metabolomic Society. 2014;10(1):63–76. Epub 2014/02/11. 10.1007/s11306-013-0552-7 24511307 PMC3913270

[67] Ghosh S, Indracanti N, Joshi J, Ray J, Indraganti PK. Pharmacologically induced reversible hypometabolic state mitigates radiation induced lethality in mice. Sci Rep. 2017;7(1):14900. Epub 2017/11/04. 10.1038/s41598-017-15002-7 29097738 PMC5668348

[68] Gorr TA. Hypometabolism as the ultimate defence in stress response: how the comparative approach helps understanding of medically relevant questions. Acta Physiol (Oxf). 2017;219(2):409–40. Epub 2016/07/02. 10.1111/apha.12747 . 27364602

[69] Vincenzi F, Pasquini S, Borea PA, Varani K. Targeting Adenosine Receptors: A Potential Pharmacological Avenue for Acute and Chronic Pain. Int J Mol Sci. 2020;21(22). Epub 2020/11/22. 10.3390/ijms21228710 33218074 PMC7698931

[70] Silvani A, Cerri M, Zoccoli G, Swoap SJ. Is Adenosine Action Common Ground for NREM Sleep, Torpor, and Other Hypometabolic States? Physiology (Bethesda). 2018;33(3):182–96. Epub 2018/04/05. 10.1152/physiol.00007.2018 29616880 PMC5966658

[71] Riteau N, Baron L, Villeret B, Guillou N, Savigny F, Ryffel B, et al. ATP release and purinergic signaling: a common pathway for particle-mediated inflammasome activation. Cell Death Dis. 2012;3:e403. 10.1038/cddis.2012.144 23059822 PMC3481132

[72] Pelegrin P. P2X7 receptor and the NLRP3 inflammasome: Partners in crime. Biochemical pharmacology. 2020:114385. Epub 2020/12/29. 10.1016/j.bcp.2020.114385 . 33359010

[73] Kozumbo WJ, Calabrese EJ. Two decades (1998–2018) of research Progress on Hormesis: advancing biological understanding and enabling novel applications. J Cell Commun Signal. 2019;13(3):273–5. Epub 2019/04/19. 10.1007/s12079-019-00517-7 30997652 PMC6732134

[74] Merry TL, Ristow M. Mitohormesis in exercise training. Free radical biology & medicine. 2016;98:123–30. Epub 2015/12/15. 10.1016/j.freeradbiomed.2015.11.032 . 26654757

[75] Davies KJ. Adaptive homeostasis. Molecular aspects of medicine. 2016;49:1–7. Epub 2016/04/27. 10.1016/j.mam.2016.04.007 27112802 PMC4868097

[76] Eagleson KL, Villaneuva M, Southern RM, Levitt P. Proteomic and mitochondrial adaptations to early-life stress are distinct in juveniles and adults. Neurobiol Stress. 2020;13:100251. Epub 2020/12/22. 10.1016/j.ynstr.2020.100251 33344706 PMC7739184

[77] Hurwitz BJ, Jeffery D, Arnason B, Bigley K, Coyle P, Goodin D, et al. Tolerability and safety profile of 12- to 28-week treatment with interferon beta-1b 250 and 500 microg QOD in patients with relapsing-remitting multiple sclerosis: a multicenter, randomized, double-blind, parallel-group pilot study. Clin Ther. 2008;30(6):1102–12. Epub 2008/07/22. 10.1016/j.clinthera.2008.06.013 . 18640466

[78] Shattuck EC, Muehlenbein MP. Human sickness behavior: Ultimate and proximate explanations. Am J Phys Anthropol. 2015;157(1):1–18. Epub 2015/02/03. 10.1002/ajpa.22698 . 25639499

[79] Li H, Cvejic E, Gu B, Vollmer-Conna U, Hickie I, Wakefield D, et al. Regulation of the acute sickness response by the P2X7 receptor. The Journal of infectious diseases. 2021. Epub 2021/01/21. 10.1093/infdis/jiab027 . 33471105

[80] Hand SC, Denlinger DL, Podrabsky JE, Roy R. Mechanisms of animal diapause: recent developments from nematodes, crustaceans, insects, and fish. American journal of physiology Regulatory, integrative and comparative physiology. 2016;310(11):R1193–211. Epub 2016/04/08. 10.1152/ajpregu.00250.2015 27053646 PMC4935499

[81] Schreiner P, Harrer T, Scheibenbogen C, Lamer S, Schlosser A, Naviaux RK, et al. Human Herpesvirus-6 Reactivation, Mitochondrial Fragmentation, and the Coordination of Antiviral and Metabolic Phenotypes in Myalgic Encephalomyelitis/Chronic Fatigue Syndrome. Immunohorizons. 2020;4(4):201–15. Epub 2020/04/25. 10.4049/immunohorizons.2000006 . 32327453

[82] Cheroni C, Caporale N, Testa G. Autism spectrum disorder at the crossroad between genes and environment: contributions, convergences, and interactions in ASD developmental pathophysiology. Molecular autism. 2020;11(1):69. Epub 2020/09/12. 10.1186/s13229-020-00370-1 32912338 PMC7488083

[83] Gavett BE, Stern RA, McKee AC. Chronic traumatic encephalopathy: a potential late effect of sport-related concussive and subconcussive head trauma. Clinics in sports medicine. 2011;30(1):179–88, xi. Epub 2010/11/16. 10.1016/j.csm.2010.09.007 21074091 PMC2995699

[84] Kv Frisch. Über einen Schreckstoff der Fischhaut und seine biologische Bedeutung. Zeitschrift für vergleichende Physiologie. 1942;29(1–2):46–145.

[85] Kv Frisch. Zur psychologie des fisch-schwarmes. Naturwissenschaften. 1938;26(37):601–6.

[86] Brown GE, Adrian JC, Smyth E, Leet H, Brennan S. Ostariophysan alarm pheromones: laboratory and field tests of the functional significance of nitrogen oxides. Journal of Chemical Ecology. 2000;26(1):139–54.

[87] Mathuru AS, Kibat C, Cheong WF, Shui G, Wenk MR, Friedrich RW, et al. Chondroitin fragments are odorants that trigger fear behavior in fish. Curr Biol. 2012;22(6):538–44. Epub 2012/03/01. 10.1016/j.cub.2012.01.061 . 22365850

[88] Chivers DP, Wisenden BD, Hindman CJ, Michalak TA, Kusch RC, Kaminskyj SG, et al. Epidermal ’alarm substance’ cells of fishes maintained by non-alarm functions: possible defence against pathogens, parasites and UVB radiation. Proc Biol Sci. 2007;274(1625):2611–9. Epub 2007/08/10. 10.1098/rspb.2007.0709 17686729 PMC2275884

[89] Chia JSM, Wall ES, Wee CL, Rowland TAJ, Cheng RK, Cheow K, et al. Bacteria evoke alarm behaviour in zebrafish. Nat Commun. 2019;10(1):3831. Epub 2019/08/25. 10.1038/s41467-019-11608-9 31444339 PMC6707203

[90] Tanaka K, Gilroy S, Jones AM, Stacey G. Extracellular ATP signaling in plants. Trends in cell biology. 2010;20(10):601–8. Epub 2010/09/08. S0962-8924(10)00150-9 [pii] 10.1016/j.tcb.2010.07.005 . 20817461 PMC4864069

[91] Fan KQ, Li YY, Wang HL, Mao XT, Guo JX, Wang F, et al. Stress-Induced Metabolic Disorder in Peripheral CD4(+) T Cells Leads to Anxiety-like Behavior. Cell. 2019;179(4):864–79 e19. Epub 2019/11/02. 10.1016/j.cell.2019.10.001 . 31675497

[92] Makrides V, Camargo SM, Verrey F. Transport of amino acids in the kidney. Comprehensive Physiology. 2014;4(1):367–403. Epub 2014/04/03. 10.1002/cphy.c130028 . 24692143

[93] Kim Y, Sundrud MS, Zhou C, Edenius M, Zocco D, Powers K, et al. Aminoacyl-tRNA synthetase inhibition activates a pathway that branches from the canonical amino acid response in mammalian cells. Proceedings of the National Academy of Sciences of the United States of America. 2020;117(16):8900–11. Epub 2020/04/08. 10.1073/pnas.1913788117 32253314 PMC7183223

[94] Peng W, Robertson L, Gallinetti J, Mejia P, Vose S, Charlip A, et al. Surgical stress resistance induced by single amino acid deprivation requires Gcn2 in mice. Science translational medicine. 2012;4(118):118ra11. Epub 2012/01/27. 10.1126/scitranslmed.3002629 22277968 PMC3535286

[95] Barcena C, Quiros PM, Durand S, Mayoral P, Rodriguez F, Caravia XM, et al. Methionine Restriction Extends Lifespan in Progeroid Mice and Alters Lipid and Bile Acid Metabolism. Cell reports. 2018;24(9):2392–403. Epub 2018/08/30. 10.1016/j.celrep.2018.07.089 30157432 PMC6130051

[96] Naviaux RK. Incomplete Healing as a Cause of Aging: The Role of Mitochondria and the Cell Danger Response. Biology (Basel). 2019;8(2). Epub 2019/05/15. 10.3390/biology8020027 . 31083530 PMC6627909

[97] Frye RE, Melnyk S, Macfabe DF. Unique acyl-carnitine profiles are potential biomarkers for acquired mitochondrial disease in autism spectrum disorder. Translational psychiatry. 2013;3:e220. 10.1038/tp.2012.143 23340503 PMC3566723

[98] Adams JB, Audhya T, Geis E, Gehn E, Fimbres V, Pollard EL, et al. Comprehensive Nutritional and Dietary Intervention for Autism Spectrum Disorder-A Randomized, Controlled 12-Month Trial. Nutrients. 2018;10(3). Epub 2018/03/23. 10.3390/nu10030369 29562612 PMC5872787

[99] Hendren RL, James SJ, Widjaja F, Lawton B, Rosenblatt A, Bent S. Randomized, Placebo-Controlled Trial of Methyl B12 for Children with Autism. Journal of child and adolescent psychopharmacology. 2016;26(9):774–83. Epub 2016/02/20. 10.1089/cap.2015.0159 . 26889605

[100] Zonulin Fasano A. and its regulation of intestinal barrier function: the biological door to inflammation, autoimmunity, and cancer. Physiol Rev. 2011;91(1):151–75. Epub 2011/01/21. 10.1152/physrev.00003.2008 . 21248165

[101] McKay DM, Mancini NL, Shearer J, Shutt T. Perturbed mitochondrial dynamics, an emerging aspect of epithelial-microbe interactions. American journal of physiology Gastrointestinal and liver physiology. 2020;318(4):G748–G62. Epub 2020/03/03. 10.1152/ajpgi.00031.2020 . 32116020

[102] Stucki D, Stahl W. Carbon monoxide—beyond toxicity? Toxicol Lett. 2020;333:251–60. Epub 2020/08/30. 10.1016/j.toxlet.2020.08.010 . 32860873

[103] Grabowska-Polanowska B, Skowron M, Miarka P, Pietrzycka A, Sliwka I. The application of chromatographic breath analysis in the search of volatile biomarkers of chronic kidney disease and coexisting type 2 diabetes mellitus. Journal of chromatography B, Analytical technologies in the biomedical and life sciences. 2017;1060:103–10. Epub 2017/06/13. 10.1016/j.jchromb.2017.05.030 . 28605624

[104] Cooper CE, Brown GC. The inhibition of mitochondrial cytochrome oxidase by the gases carbon monoxide, nitric oxide, hydrogen cyanide and hydrogen sulfide: chemical mechanism and physiological significance. Journal of bioenergetics and biomembranes. 2008;40(5):533–9. Epub 2008/10/08. 10.1007/s10863-008-9166-6 . 18839291

[105] Jiang F, Zhang Y, Dusting GJ. NADPH oxidase-mediated redox signaling: roles in cellular stress response, stress tolerance, and tissue repair. Pharmacological reviews. 2011;63(1):218–42. Epub 2011/01/14. 10.1124/pr.110.002980 . 21228261

[106] Gelmont D, Stein RA, Mead JF. Isoprene-the main hydrocarbon in human breath. Biochemical and biophysical research communications. 1981;99(4):1456–60. Epub 1981/04/30. 10.1016/0006-291x(81)90782-8 . 7259787

[107] King J, Koc H, Unterkofler K, Mochalski P, Kupferthaler A, Teschl G, et al. Physiological modeling of isoprene dynamics in exhaled breath. J Theor Biol. 2010;267(4):626–37. Epub 2010/09/28. 10.1016/j.jtbi.2010.09.028 . 20869370

[108] Fradejas N, Carlson BA, Rijntjes E, Becker NP, Tobe R, Schweizer U. Mammalian Trit1 is a tRNA([Ser]Sec)-isopentenyl transferase required for full selenoprotein expression. The Biochemical journal. 2013;450(2):427–32. Epub 2013/01/08. 10.1042/BJ20121713 . 23289710

[109] Berthelot K, Estevez Y, Deffieux A, Peruch F. Isopentenyl diphosphate isomerase: A checkpoint to isoprenoid biosynthesis. Biochimie. 2012;94(8):1621–34. Epub 2012/04/17. 10.1016/j.biochi.2012.03.021 . 22503704

[110] Morello G, Guarnaccia M, Spampinato AG, La Cognata V, D’Agata V, Cavallaro S. Copy Number Variations in Amyotrophic Lateral Sclerosis: Piecing the Mosaic Tiles Together through a Systems Biology Approach. Molecular neurobiology. 2018;55(2):1299–322. Epub 2017/01/26. 10.1007/s12035-017-0393-x 28120152 PMC5820374

[111] Nakamura K, Mori F, Tanji K, Miki Y, Yamada M, Kakita A, et al. Isopentenyl diphosphate isomerase, a cholesterol synthesizing enzyme, is localized in Lewy bodies. Neuropathology: official journal of the Japanese Society of Neuropathology. 2015;35(5):432–40. Epub 2015/05/08. 10.1111/neup.12204 . 25950736

[112] Sharon C, Baranwal S, Patel NJ, Rodriguez-Agudo D, Pandak WM, Majumdar AP, et al. Inhibition of insulin-like growth factor receptor/AKT/mammalian target of rapamycin axis targets colorectal cancer stem cells by attenuating mevalonate-isoprenoid pathway in vitro and in vivo. Oncotarget. 2015;6(17):15332–47. Epub 2015/04/22. 10.18632/oncotarget.3684 25895029 PMC4558155

[113] Muhlebner A, Bongaarts A, Sarnat HB, Scholl T, Aronica E. New insights into a spectrum of developmental malformations related to mTOR dysregulations: challenges and perspectives. J Anat. 2019;235(3):521–42. Epub 2019/03/23. 10.1111/joa.12956 30901081 PMC6704243

[114] Wang H, Sarikonda G, Puan KJ, Tanaka Y, Feng J, Giner JL, et al. Indirect stimulation of human Vgamma2Vdelta2 T cells through alterations in isoprenoid metabolism. J Immunol. 2011;187(10):5099–113. Epub 2011/10/21. 10.4049/jimmunol.1002697 22013129 PMC3326638

[115] Warner GJ, Berry MJ, Moustafa ME, Carlson BA, Hatfield DL, Faust JR. Inhibition of selenoprotein synthesis by selenocysteine tRNA[Ser]Sec lacking isopentenyladenosine. The Journal of biological chemistry. 2000;275(36):28110–9. Epub 2000/05/24. 10.1074/jbc.M001280200 . 10821829

[116] Smith D, Spanel P, Enderby B, Lenney W, Turner C, Davies SJ. Isoprene levels in the exhaled breath of 200 healthy pupils within the age range 7–18 years studied using SIFT-MS. Journal of breath research. 2010;4(1):017101. Epub 2009/12/18. 10.1088/1752-7155/4/1/017101 . 21386206

[117] Karl T, Prazeller P, Mayr D, Jordan A, Rieder J, Fall R, et al. Human breath isoprene and its relation to blood cholesterol levels: new measurements and modeling. J Appl Physiol (1985). 2001;91(2):762–70. Epub 2001/07/18. 10.1152/jappl.2001.91.2.762 . 11457792

[118] Hill SD, Wagner EA, Shedlarski JG Jr., Sears SP. Diurnal cortisol and temperature variation of normal and autistic children. 10.1002/dev.420100612 . 1977;10(6):579–83. 563824

[119] Stussman B, Williams A, Snow J, Gavin A, Scott R, Nath A, et al. Characterization of Post-exertional Malaise in Patients With Myalgic Encephalomyelitis/Chronic Fatigue Syndrome. Front Neurol. 2020;11:1025. Epub 2020/10/20. 10.3389/fneur.2020.01025 33071931 PMC7530890

[120] Curran LK, Newschaffer CJ, Lee LC, Crawford SO, Johnston MV, Zimmerman AW. Behaviors associated with fever in children with autism spectrum disorders. Pediatrics. 2007;120(6):e1386–92. 10.1542/peds.2007-0360 . 18055656

[121] Naviaux RK. Antipurinergic therapy for autism-An in-depth review. Mitochondrion. 2018;43:1–15. Epub 2017/12/19. 10.1016/j.mito.2017.12.007 . 29253638

[122] Burnstock G. Introduction to Purinergic Signalling in the Brain. Advances in experimental medicine and biology. 2020;1202:1–12. Epub 2020/02/09. 10.1007/978-3-030-30651-9_1 . 32034706

[123] Cheffer A, Castillo ARG, Correa-Velloso J, Goncalves MCB, Naaldijk Y, Nascimento IC, et al. Purinergic system in psychiatric diseases. Molecular psychiatry. 2018;23(1):94–106. Epub 2017/09/28. 10.1038/mp.2017.188 . 28948971

[124] Naviaux RK. Metabolic features and regulation of the healing cycle-A new model for chronic disease pathogenesis and treatment. Mitochondrion. 2019;46:278–97. Epub 2018/08/14. 10.1016/j.mito.2018.08.001 . 30099222

[125] Naviaux RK, Naviaux JC, Li K, Bright AT, Alaynick WA, Wang L, et al. Metabolic features of chronic fatigue syndrome. Proceedings of the National Academy of Sciences of the United States of America. 2016;113(37):E5472–80. 10.1073/pnas.1607571113 27573827 PMC5027464

[126] Naviaux RK, Naviaux JC, Li K, Wang L, Monk JM, Bright AT, et al. Metabolic features of Gulf War illness. PloS one. 2019;14(7):e0219531. Epub 2019/07/28. 10.1371/journal.pone.0219531 . 31348786 PMC6660083

[127] Illes P, Verkhratsky A, Tang Y. Pathological ATPergic Signaling in Major Depression and Bipolar Disorder. Frontiers in molecular neuroscience. 2019;12:331. Epub 2020/02/23. 10.3389/fnmol.2019.00331 32076399 PMC7006450

[128] Illes P, Rubini P, Ulrich H, Zhao Y, Tang Y. Regulation of Microglial Functions by Purinergic Mechanisms in the Healthy and Diseased CNS. Cells. 2020;9(5). Epub 2020/05/06. 10.3390/cells9051108 PubMed Central PMCID: PMC7290360. 32365642 PMC7290360

[129] Frye RE. Mitochondrial Dysfunction in Autism Spectrum Disorder: Unique Abnormalities and Targeted Treatments. Semin Pediatr Neurol. 2020;35:100829. Epub 2020/09/08. 10.1016/j.spen.2020.100829 . 32892956

[130] Fasano A, Hill I. Serum Zonulin, Gut Permeability, and the Pathogenesis of Autism Spectrum Disorders: Cause, Effect, or an Epiphenomenon? The Journal of pediatrics. 2017;188:15–7. Epub 2017/06/19. 10.1016/j.jpeds.2017.05.038 . 28624097

[131] Saurman V, Margolis KG, Luna RA. Autism Spectrum Disorder as a Brain-Gut-Microbiome Axis Disorder. Digestive diseases and sciences. 2020;65(3):818–28. Epub 2020/02/15. 10.1007/s10620-020-06133-5 32056091 PMC7580230

[132] Theoharides TC, Kavalioti M, Tsilioni I. Mast Cells, Stress, Fear and Autism Spectrum Disorder. Int J Mol Sci. 2019;20(15). Epub 2019/07/28. 10.3390/ijms20153611 31344805 PMC6696098

[133] Yoshida K, Ito MA, Sato N, Obayashi K, Yamamoto K, Koizumi S, et al. Extracellular ATP Augments Antigen-Induced Murine Mast Cell Degranulation and Allergic Responses via P2X4 Receptor Activation. J Immunol. 2020;204(12):3077–85. Epub 2020/05/03. 10.4049/jimmunol.1900954 . 32358018

[134] Jyonouchi H, Geng L. Associations between Monocyte and T Cell Cytokine Profiles in Autism Spectrum Disorders: Effects of Dysregulated Innate Immune Responses on Adaptive Responses to Recall Antigens in a Subset of ASD Children. Int J Mol Sci. 2019;20(19). Epub 2019/09/27. 10.3390/ijms20194731 31554204 PMC6801811

[135] Chavez CE, Oyarzun JE, Avendano BC, Mellado LA, Inostroza CA, Alvear TF, et al. The Opening of Connexin 43 Hemichannels Alters Hippocampal Astrocyte Function and Neuronal Survival in Prenatally LPS-Exposed Adult Offspring. Frontiers in cellular neuroscience. 2019;13:460. Epub 2019/11/05. 10.3389/fncel.2019.00460 31680871 PMC6797550

[136] Vargas DL, Nascimbene C, Krishnan C, Zimmerman AW, Pardo CA. Neuroglial activation and neuroinflammation in the brain of patients with autism. Annals of neurology. 2005;57(1):67–81. 10.1002/ana.20315 . 15546155

[137] Grkovic I, Drakulic D, Martinovic J, Mitrovic N. Role of Ectonucleotidases in Synapse Formation During Brain Development: Physiological and Pathological Implications. Curr Neuropharmacol. 2019;17(1):84–98. Epub 2017/05/20. 10.2174/1570159X15666170518151541 28521702 PMC6341498

[138] Smith M, Flodman PL, Gargus JJ, Simon MT, Verrell K, Haas R, et al. Mitochondrial and ion channel gene alterations in autism. Biochimica et biophysica acta. 2012;1817(10):1796–802. 10.1016/j.bbabio.2012.04.004 22538295 PMC3423964

[139] Palmieri L, Papaleo V, Porcelli V, Scarcia P, Gaita L, Sacco R, et al. Altered calcium homeostasis in autism-spectrum disorders: evidence from biochemical and genetic studies of the mitochondrial aspartate/glutamate carrier AGC1. Molecular psychiatry. 2010;15(1):38–52. Epub 2008/07/09. 10.1038/mp.2008.63 . 18607376

[140] Martorell A, Wellmann M, Guiffa F, Fuenzalida M, Bonansco C. P2Y1 receptor inhibition rescues impaired synaptic plasticity and astroglial Ca(2+)-dependent activity in the epileptic hippocampus. Neurobiology of disease. 2020;146:105132. Epub 2020/10/14. 10.1016/j.nbd.2020.105132 . 33049315

[141] Ryu JK, Choi HB, Hatori K, Heisel RL, Pelech SL, McLarnon JG, et al. Adenosine triphosphate induces proliferation of human neural stem cells: Role of calcium and p70 ribosomal protein S6 kinase. J Neurosci Res. 2003;72(3):352–62. Epub 2003/04/15. 10.1002/jnr.10507 . 12692902

[142] Sacco R, Gabriele S, Persico AM. Head circumference and brain size in autism spectrum disorder: A systematic review and meta-analysis. Psychiatry research. 2015;234(2):239–51. Epub 2015/10/13. 10.1016/j.pscychresns.2015.08.016 . 26456415

[143] Dehorter N, Del Pino I. Shifting Developmental Trajectories During Critical Periods of Brain Formation. Frontiers in cellular neuroscience. 2020;14:283. Epub 2020/11/03. 10.3389/fncel.2020.00283 33132842 PMC7513795

[144] Briuglia S, Calabro M, Capra AP, Briguori S, La Rosa MA, Crisafulli C. Molecular Pathways within Autism Spectrum Disorder Endophenotypes. J Mol Neurosci. 2021. Epub 2021/01/26. 10.1007/s12031-020-01782-7 . 33492615

[145] Horvath G, Otrokocsi L, Beko K, Baranyi M, Kittel A, Fritz-Ruenes PA, et al. P2X7 Receptors Drive Poly(I:C) Induced Autism-like Behavior in Mice. The Journal of neuroscience: the official journal of the Society for Neuroscience. 2019;39(13):2542–61. Epub 2019/01/27. 10.1523/JNEUROSCI.1895-18.2019 30683682 PMC6435822

[146] Banoth B, Cassel SL. Mitochondria in innate immune signaling. Transl Res. 2018;202:52–68. Epub 2018/08/31. 10.1016/j.trsl.2018.07.014 30165038 PMC6218307

[147] Cheikhi A, Wallace C, St Croix C, Cohen C, Tang WY, Wipf P, et al. Mitochondria are a substrate of cellular memory. Free radical biology & medicine. 2019;130:528–41. Epub 2018/11/26. 10.1016/j.freeradbiomed.2018.11.028 . 30472365

[148] Bird L. Innate immunity: Linking mitochondria and microbes to inflammasomes. Nature reviews Immunology. 2012;12(4):229. 10.1038/nri3195 . 22402669

[149] Hirsch MM, Deckmann I, Santos-Terra J, Staevie GZ, Fontes-Dutra M, Carello-Collar G, et al. Effects of single-dose antipurinergic therapy on behavioral and molecular alterations in the valproic acid-induced animal model of autism. Neuropharmacology. 2020:107930. Epub 2020/01/07. 10.1016/j.neuropharm.2019.107930 . 31904357

[150] Zhang M, Li W, Niu G, Leak RK, Chen J, Zhang F. ATP induces mild hypothermia in rats but has a strikingly detrimental impact on focal cerebral ischemia. J Cereb Blood Flow Metab. 2013;33(1). Epub 2012/10/18. 10.1038/jcbfm.2012.146 23072747 PMC3597371

[151] Beijer S, Hupperets PS, van den Borne BE, Wijckmans NE, Spreeuwenberg C, van den Brandt PA, et al. Randomized clinical trial on the effects of adenosine 5’-triphosphate infusions on quality of life, functional status, and fatigue in preterminal cancer patients. J Pain Symptom Manage. 2010;40(4):520–30. 10.1016/j.jpainsymman.2010.01.023 . 20598849

[152] Lu VB, Rievaj J, O’Flaherty EA, Smith CA, Pais R, Pattison LA, et al. Adenosine triphosphate is co-secreted with glucagon-like peptide-1 to modulate intestinal enterocytes and afferent neurons. Nat Commun. 2019;10(1):1029. Epub 2019/03/06. 10.1038/s41467-019-09045-9 30833673 PMC6399286

[153] Hoey DE, Nicol M, Williams BC, Walker SW. Primary cultures of bovine inner zone adrenocortical cells secrete cortisol in response to adenosine 5’-triphosphate, adenosine 5’-diphosphate, and uridine 5’-triphosphate via a nucleotide receptor that may be coupled to two signal generation systems. Endocrinology. 1994;135(4):1553–60. Epub 1994/10/01. 10.1210/endo.135.4.7925117 . 7925117

[154] Burnstock G. Purinergic signalling in endocrine organs. Purinergic signalling. 2014;10(1):189–231. Epub 2013/11/23. 10.1007/s11302-013-9396-x 24265070 PMC3944044

[155] Siso S, Jeffrey M, Gonzalez L. Sensory circumventricular organs in health and disease. Acta neuropathologica. 2010;120(6):689–705. 10.1007/s00401-010-0743-5 . 20830478

[156] Hsiao EY, McBride SW, Hsien S, Sharon G, Hyde ER, McCue T, et al. Microbiota modulate behavioral and physiological abnormalities associated with neurodevelopmental disorders. Cell. 2013;155(7):1451–63. Epub 2013/12/10. 10.1016/j.cell.2013.11.024 24315484 PMC3897394

[157] Bakken IJ, Tveito K, Gunnes N, Ghaderi S, Stoltenberg C, Trogstad L, et al. Two age peaks in the incidence of chronic fatigue syndrome/myalgic encephalomyelitis: a population-based registry study from Norway 2008–2012. BMC Med 10.1186/s12916-014-0167-5 2014;12:167. Epub 2014/10/03. PubMed Central PMCID: PMC4189623. 25274261 PMC4189623

[158] Cameron JL, Eagleson KL, Fox NA, Hensch TK, Levitt P. Social Origins of Developmental Risk for Mental and Physical Illness. The Journal of neuroscience: the official journal of the Society for Neuroscience. 2017;37(45):10783–91. 10.1523/JNEUROSCI.1822-17.2017 . 29118206 PMC5678010

[159] Hertz-Picciotto I, Schmidt RJ, Krakowiak P. Understanding environmental contributions to autism: Causal concepts and the state of science. Autism research: official journal of the International Society for Autism Research. 2018;11(4):554–86. Epub 2018/03/25. 10.1002/aur.1938 . 29573218

