# Supplementary material for: Metabolic and behavioral features of acute hyperpurinergia and the maternal immune activation mouse model of autism spectrum disorder
Source: PLoS One. 2021 Mar 18;16(3):e0248771. doi: 10.1371/journal.pone.0248771 (PMC7971557; doi:10.1371/journal.pone.0248771)
Supplement: S1 File — (PDF) [file pone.0248771.s001.pdf]

Supplementary Information

2-19-21, *PLOS One*

Metabolic and Behavioral Features of Acute Hyperpurinergia and the MIA Mouse Model of Autism Spectrum Disorder

Zarazuela Zolkipli-Cunningham, Jane C. Naviaux, Tomohiro Nakayama, Charlotte M. Hirsch, Jonathan M. Monk, Kefeng Li, Lin Wang, Thuy P. Le, Simone Meinardi, Donald Blake, Robert K. Naviaux

Supplementary Figures and Legends

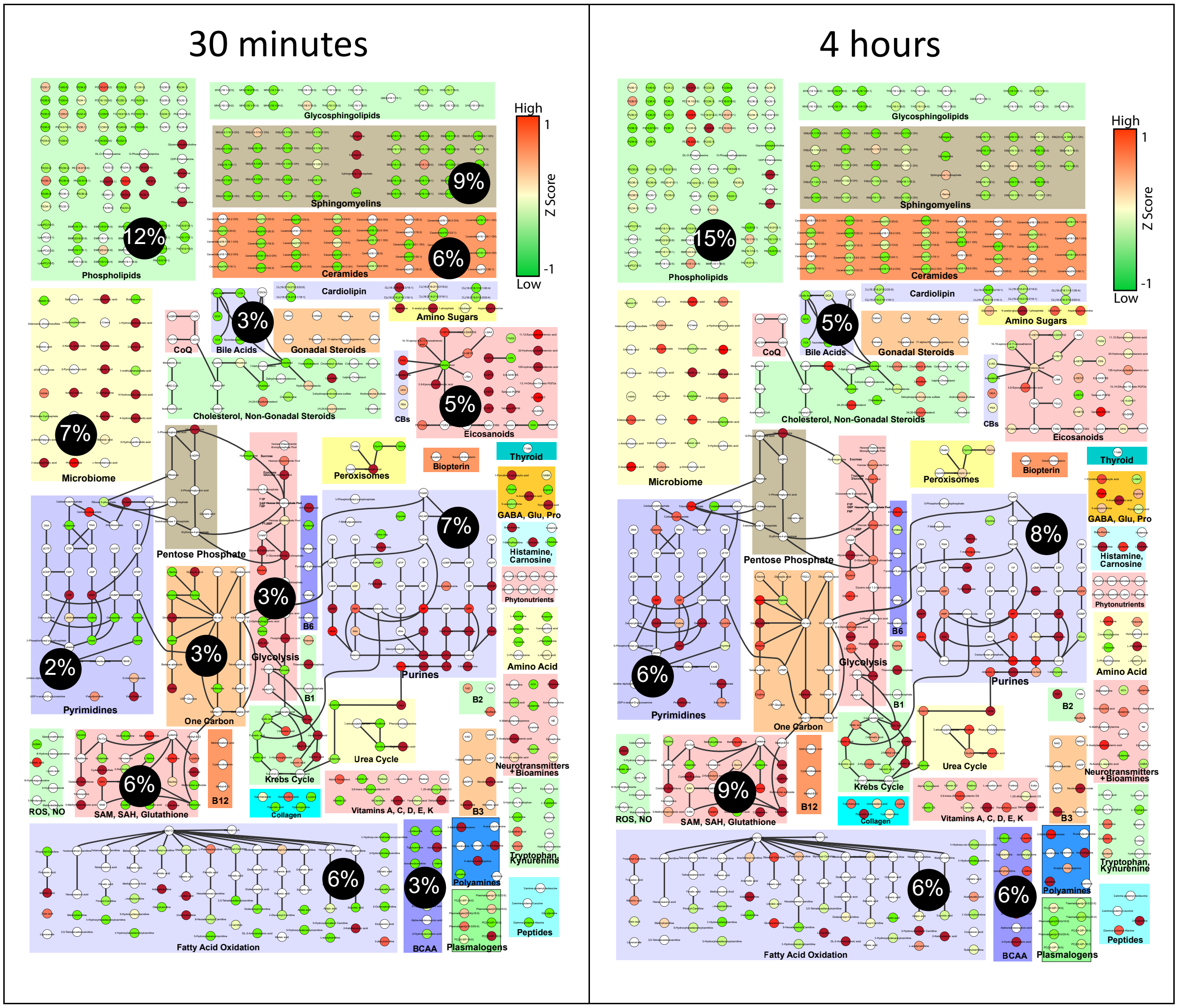

S1 Figure. Cytoscape maps of metabolic pathways changed after ATP injection. **A.** 30 minutes after injection. **B.** 4 hours after injection. Percentages in black circles indicate the fractional metabolic impact measured by VIP scores. Green indicates metabolites that were decreased. Red indicates metabolites that were increased. Dose = 0.5 μmol/g ATP IP, n = 7-8 C57Bl/6J males per group, age = 12-13 weeks.

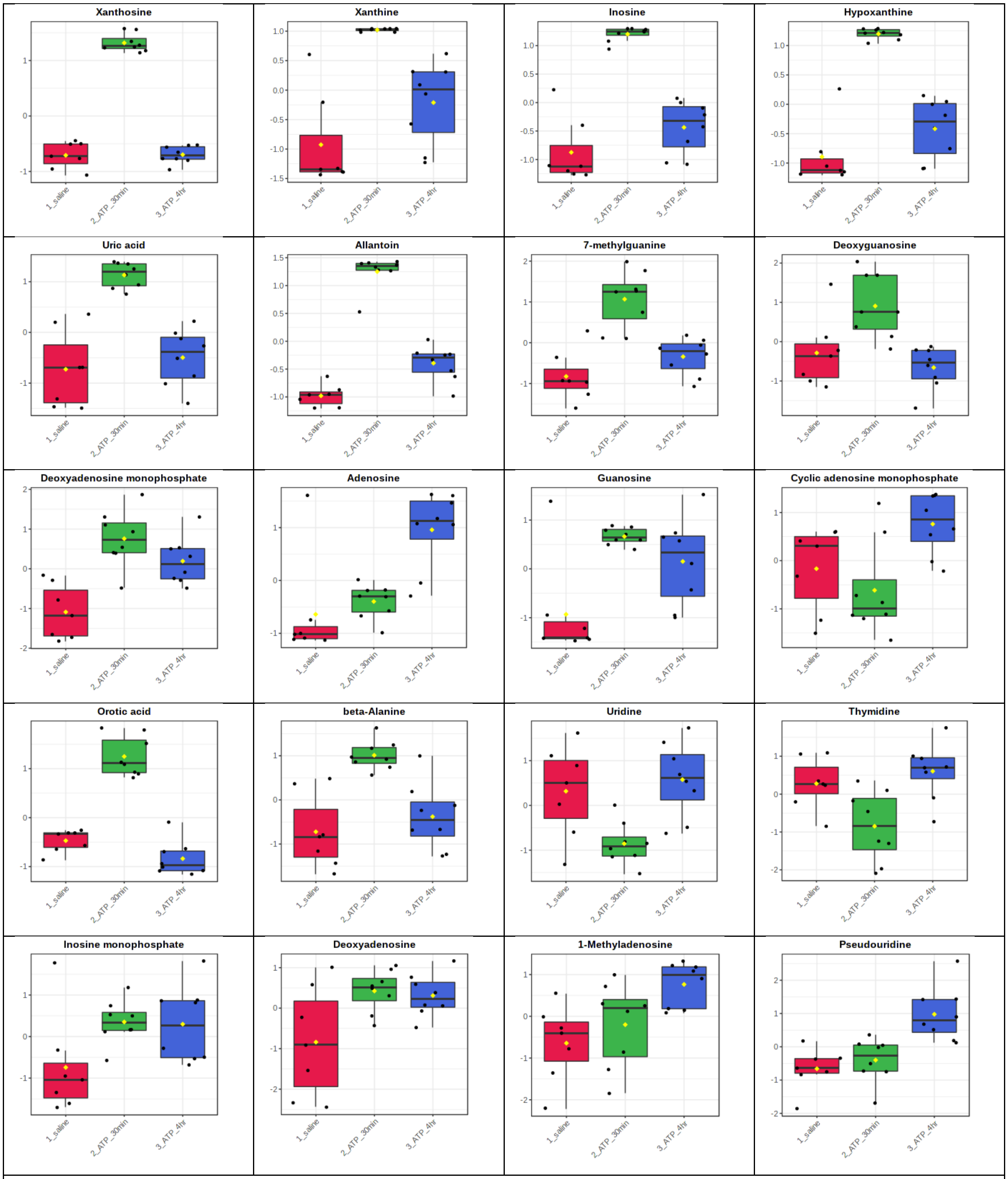

S2 Figure. **Plasma purines and pyrimidines changed after ATP injection.**

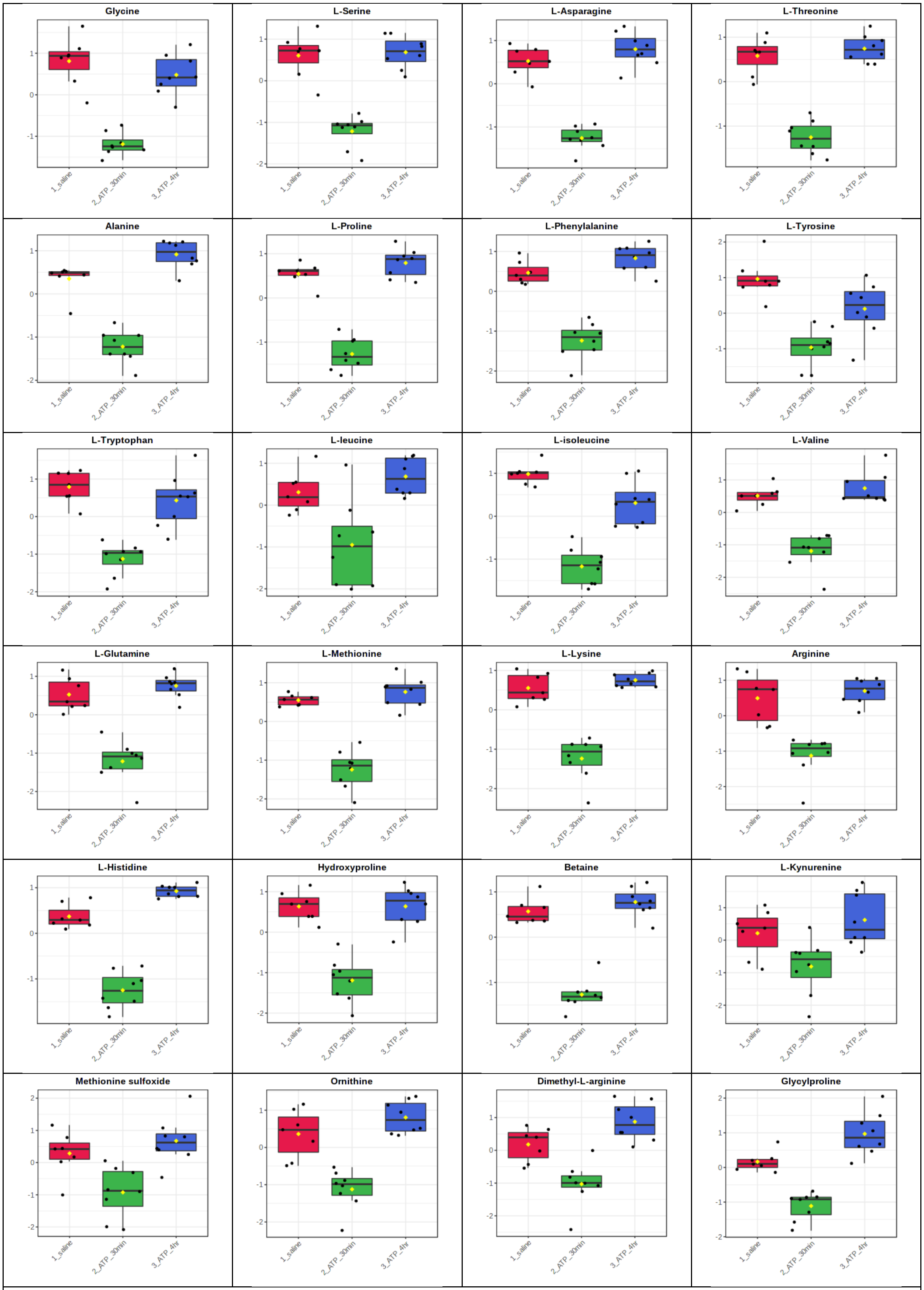

S3 Figure. Plasma amino acids were decreased 30 minutes after ATP injection.

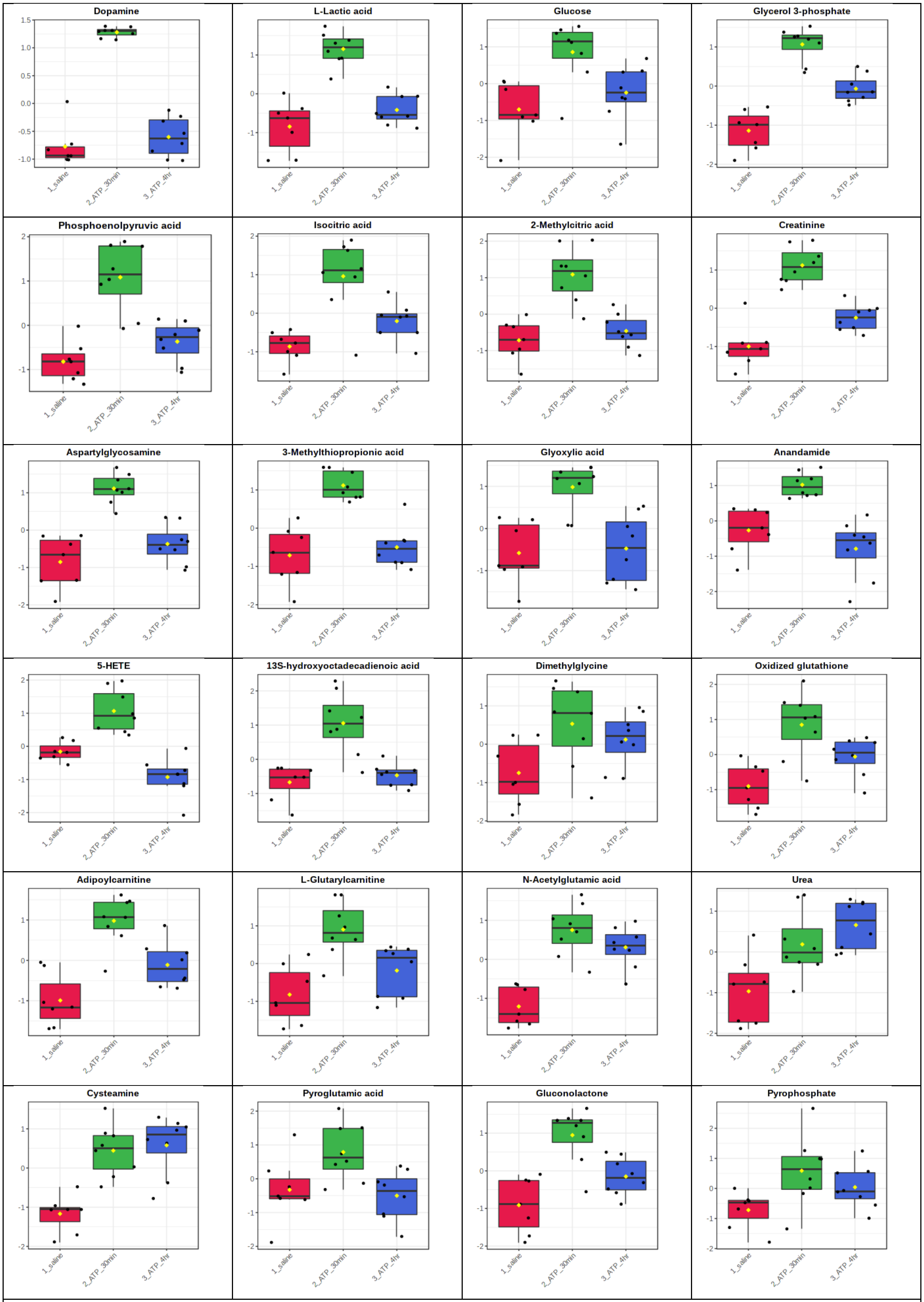

S4 Figure. k-NN supercluster of increased metabolites after ATP injection.

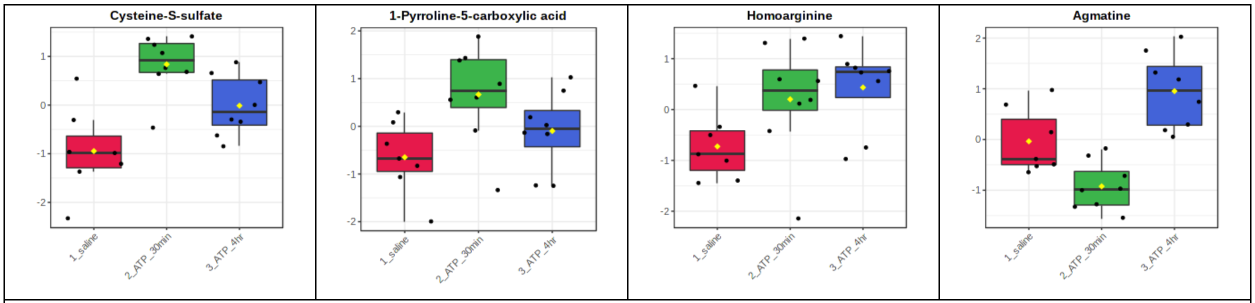

S4 Figure. k-NN supercluster of increased metabolites after ATP injection, continued.

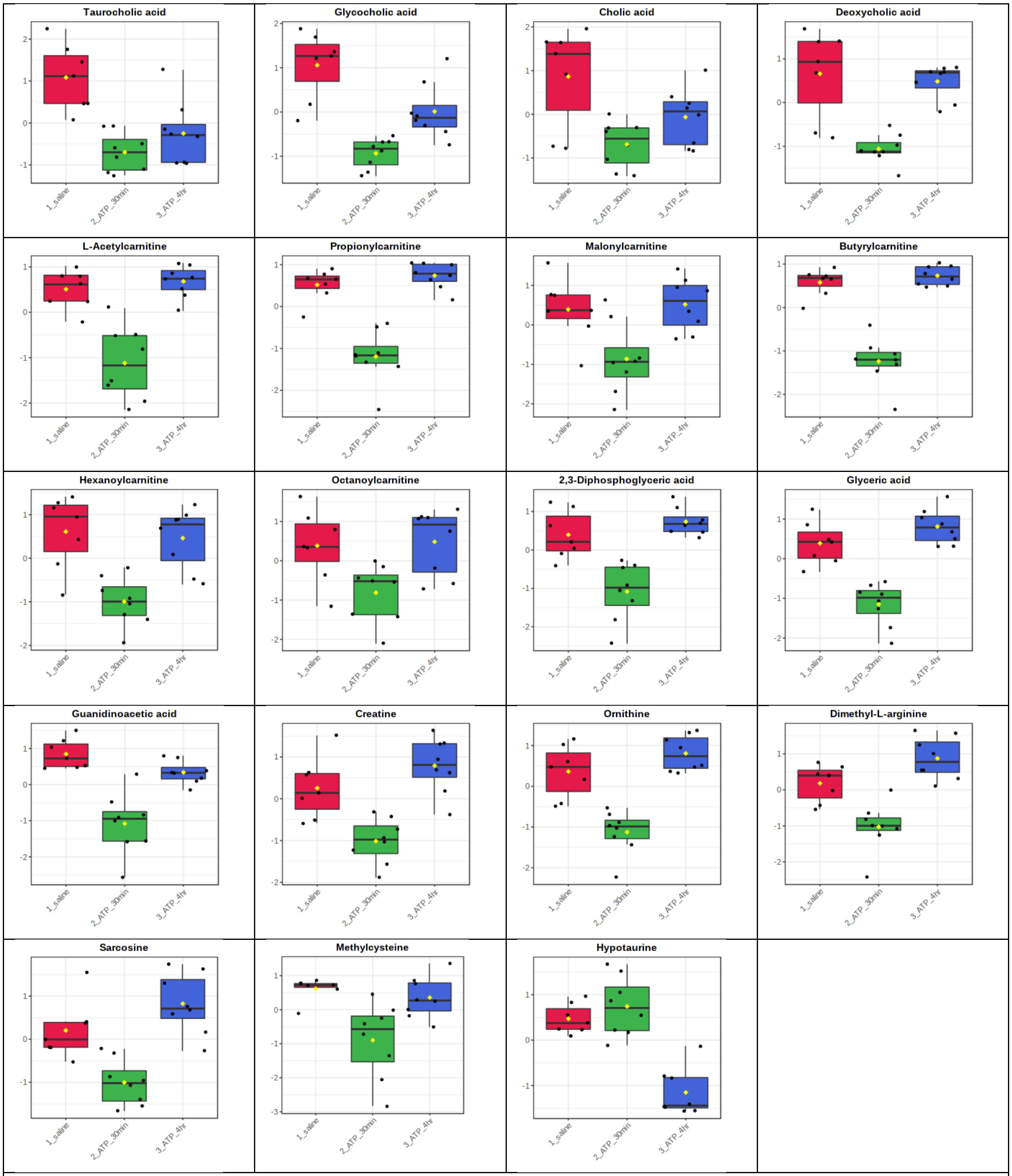

S5 Figure. *k*-NN supercluster of decreased metabolites after ATP injection.

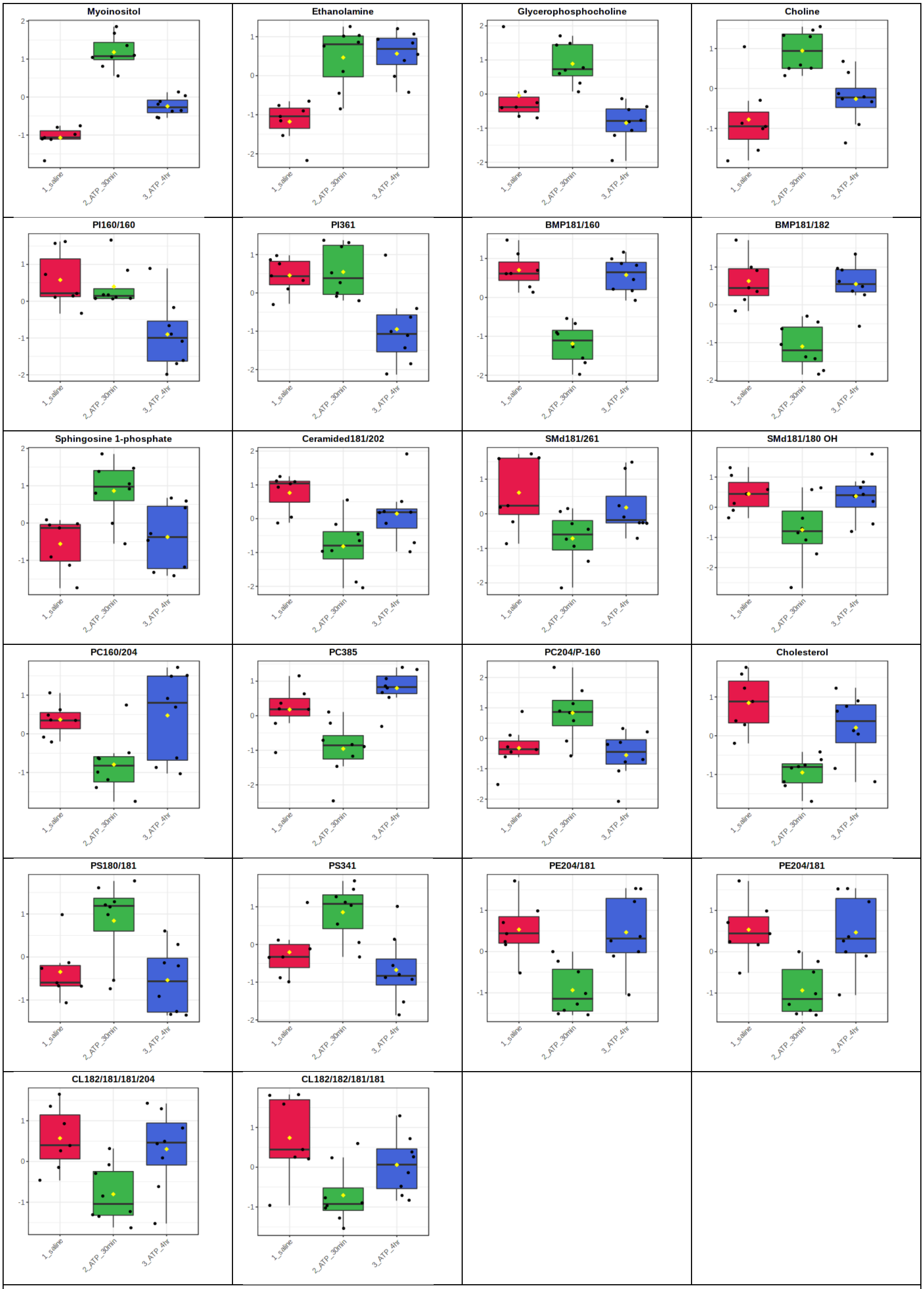

S6 Figure. Non-eicosanoid lipids and head groups changed after ATP injection.

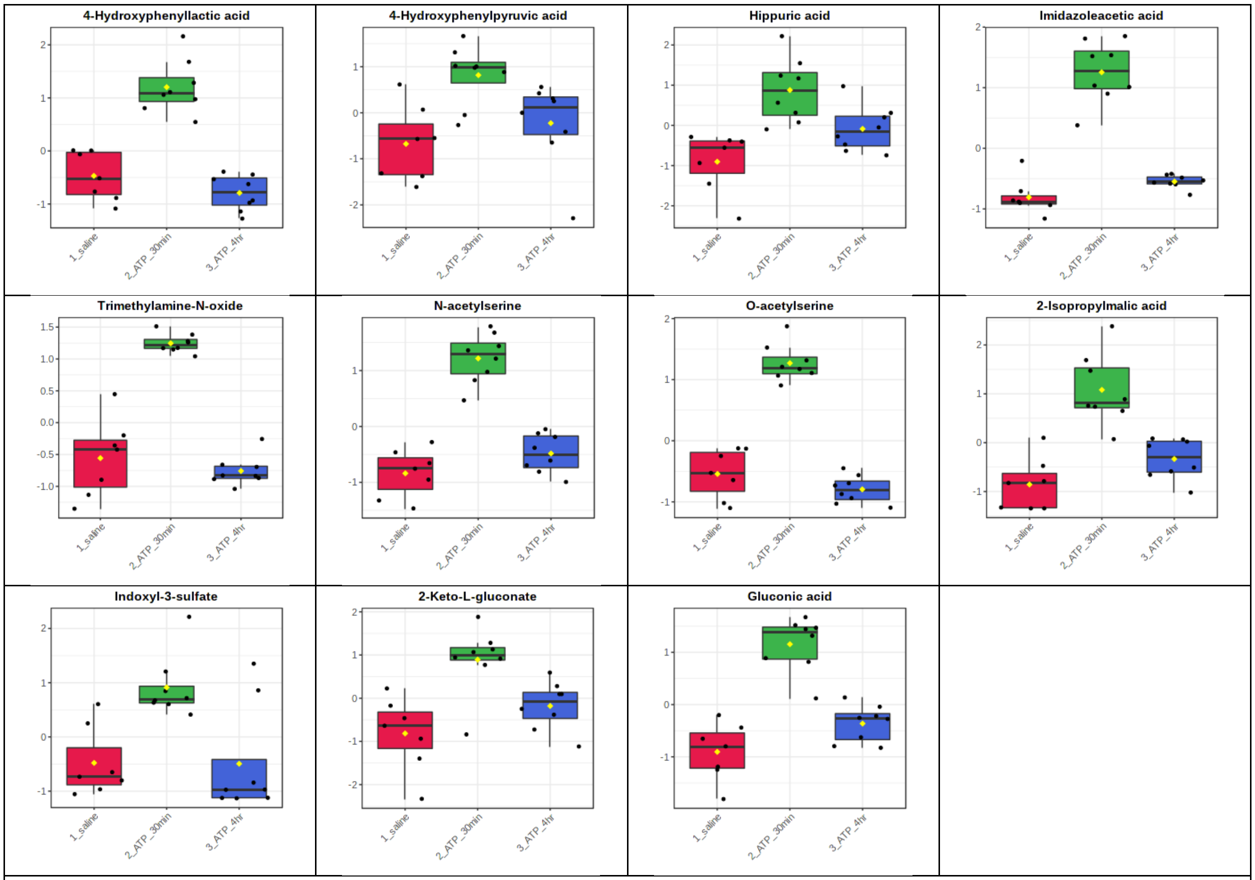

S7 Figure. Plasma microbiome metabolites increased 30 minutes after ATP injection.

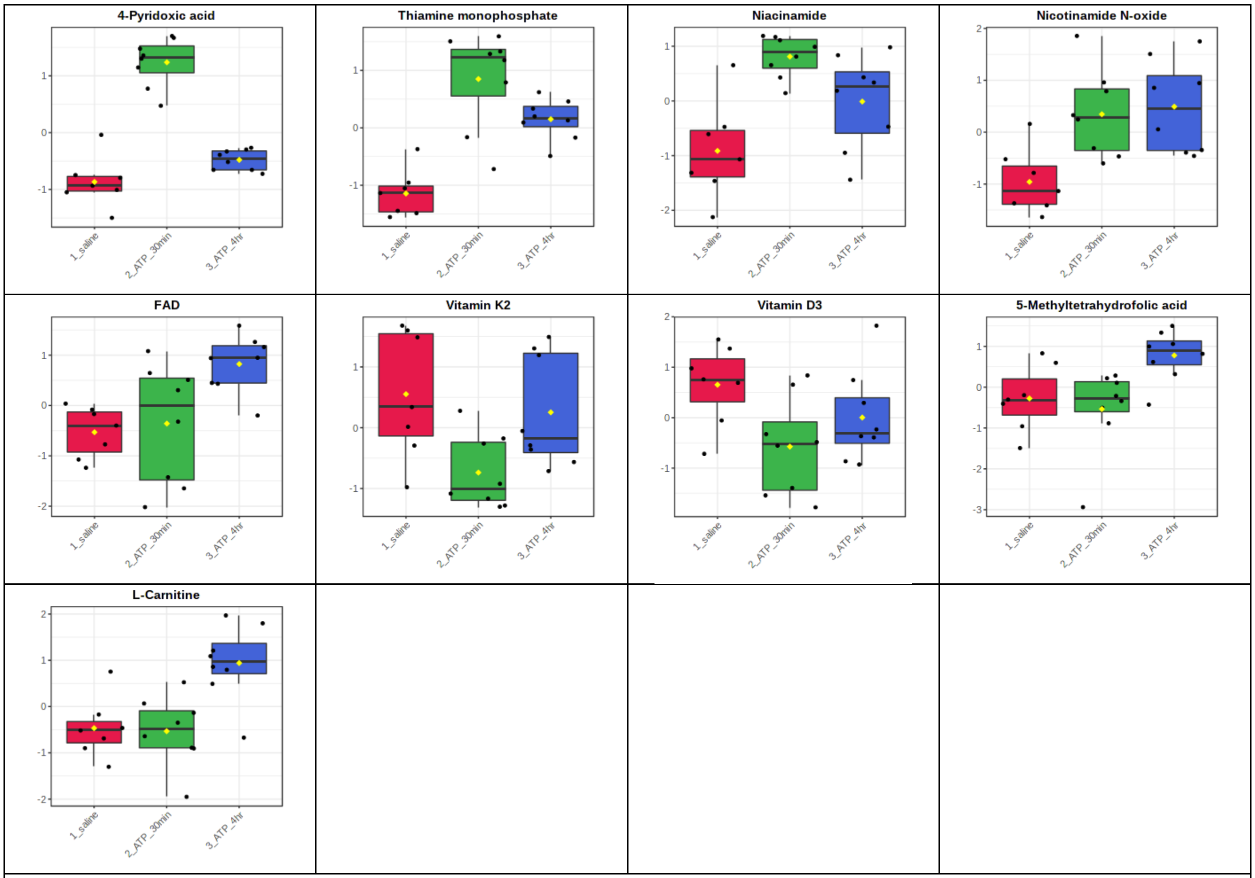

S8 Figure. Plasma vitamins and cofactors changed after ATP injection.

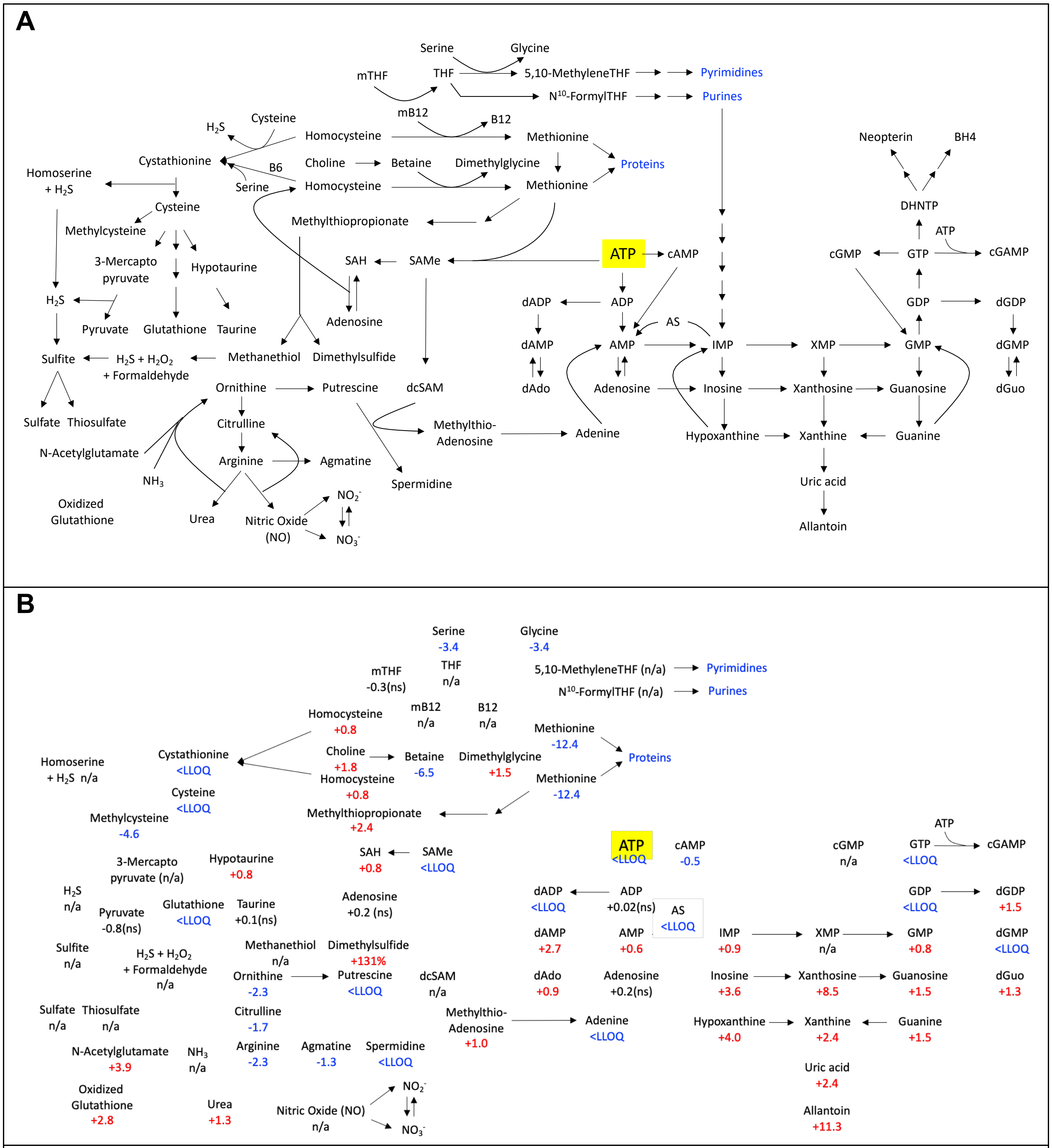

S9 Figure. Coordinated changes in purine, amino acid, methylation, sulfur, polyamine, and nitrogen metabolism produced by ATP injection. A. Flow diagram of interrelated pathways. **B.** Z-scores after 30 minutes. The ATP dose was 0.5 µmol/g IP in C57Bl/6J male mice, 12-13 weeks old, n = 7-8 per group. Red numbers indicate the Z-scores of metabolites that were increased, and blue indicates metabolites that were decreased. **Abbreviations:** DHNTP—7,8-Dihydroneopterin triphosphate; cGAMP—cyclic GMP-AMP; BH4—Tetrahydrobiopterin; LLOQ—lower limit of quantitation; n/a—metabolites that were not measured.

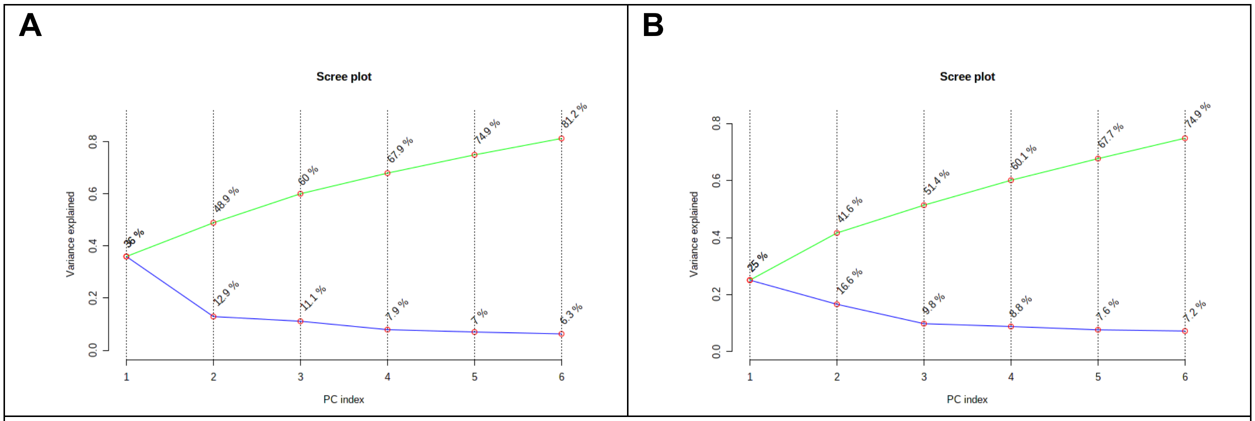

S10 Figure. Principal components analysis of metabolomic effects of ATP injection. A. 30 minutes, **B.** 4 hours after injection. The green lines indicate the cumulative proportion of phenotypic variation explained by up to 5 principal components. The blue lines indicate the fraction of variance explained by each component.

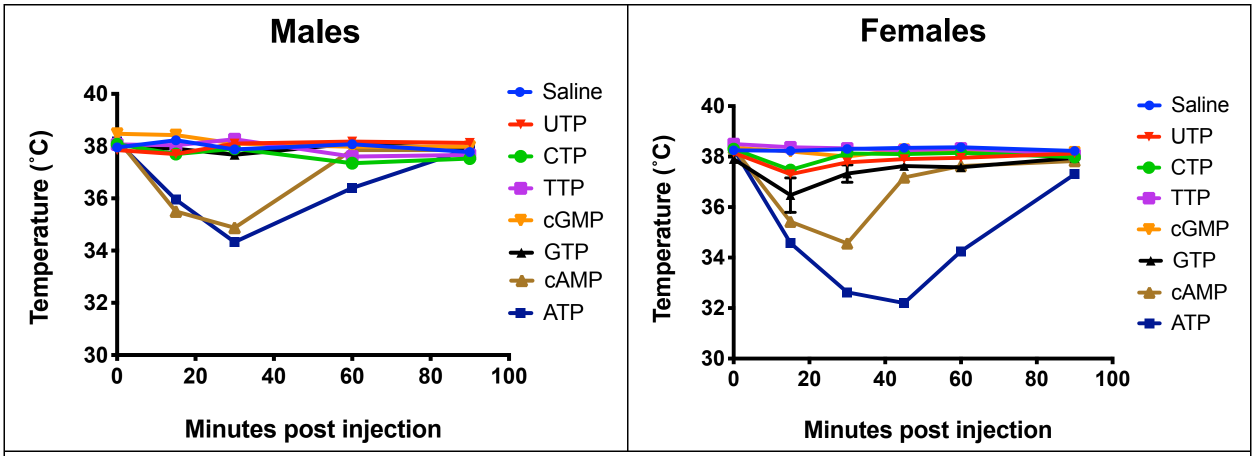

S11 Figure. Body temperature response to purines and pyrimidines. Dose of each compound was 0.5 μmol/g. n = 6 per group, 5-6 months old C57Bl/6J males and females.

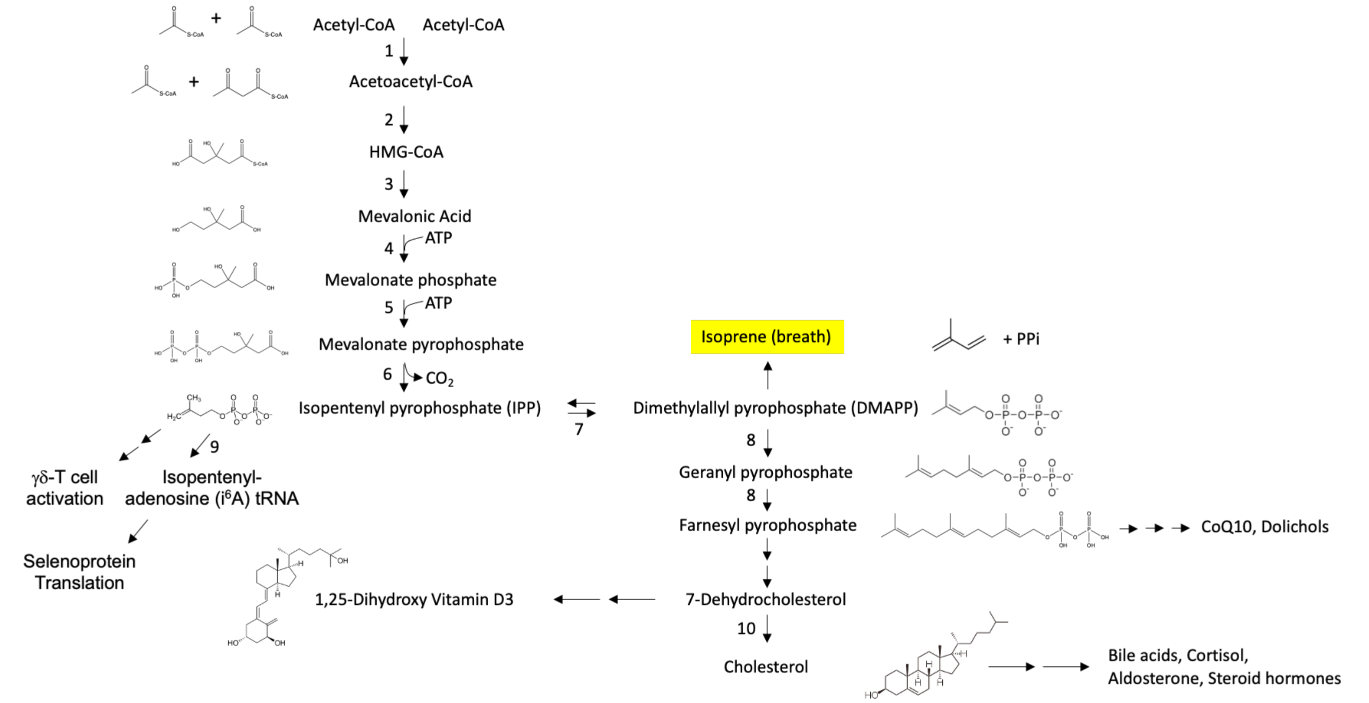

S12 Figure. Breath isoprene production. Numbered enzymatic steps: 1. Acetoacetyl-CoA transferase, 2. 3-hydroxy-3-methyl-glutaryl (HMG) CoA Synthase, 3. HMG-CoA Reductase, 4. Mevalonate kinase, 5. Phosphomevalonate kinase, 6. Mevalonate-5-pyrophosphate decarboxylase, 7. Isopentenyl-diphosphate-isomerase-1 (IDI-1), 8. Farnesyl pyrophosphate synthase (FPPS), 9. Isopentenyl pyrophosphate:tRNA isopentenyltransferase, 10. 7-Dehydrocholesterol reductase.
